# Neural synchronization and intervention in pre‐readers who later on develop dyslexia

**DOI:** 10.1111/ejn.15894

**Published:** 2022-12-23

**Authors:** Shauni Van Herck, Maria Economou, Femke Vanden Bempt, Toivo Glatz, Pol Ghesquière, Maaike Vandermosten, Jan Wouters

**Affiliations:** ^1^ Research Group ExpORL, Department of Neurosciences KU Leuven Leuven Belgium; ^2^ Parenting and Special Education Research Unit, Faculty of Psychology and Educational Sciences KU Leuven Leuven Belgium; ^3^ Leuven Brain Institute KU Leuven Leuven Belgium; ^4^ Leuven Child & Youth Institute (L‐C&Y) KU Leuven Leuven Belgium; ^5^ Institute of Public Health Charité – Universitätsmedizin Berlin, Corporate Member of Freie Universität Berlin and Humboldt‐Universität zu Berlin Berlin Germany

**Keywords:** Auditory Steady‐State Response, auditory temporal processing, phonics‐based training, reading development, speech envelope enhancement

## Abstract

A growing number of studies has investigated temporal processing deficits in dyslexia. These studies largely focus on neural synchronization to speech. However, the importance of rise times for neural synchronization is often overlooked. Furthermore, targeted interventions, phonics‐based and auditory, are being developed, but little is known about their impact. The current study investigated the impact of a 12‐week tablet‐based intervention. Children at risk for dyslexia received phonics‐based training, either with (*n* = 31) or without (*n* = 31) auditory training, or engaged in active control training (*n* = 29). Additionally, neural synchronization and processing of rise times was longitudinally investigated in children with dyslexia (*n* = 26) and typical readers (*n* = 52) from pre‐reading (5 years) to beginning reading age (7 years). The three time points in the longitudinal study correspond to intervention pre‐test, post‐test and consolidation, approximately 1 year after completing the intervention. At each time point neural synchronization was measured to sinusoidal stimuli and pulsatile stimuli with shortened rise times at syllable (4 Hz) and phoneme rates (20 Hz). Our results revealed no impact on neural synchronization at syllable and phoneme rate of the phonics‐based and auditory training. However, we did reveal atypical hemispheric specialization at both syllable and phoneme rates in children with dyslexia. This was detected even before the onset of reading acquisition, pointing towards a possible causal rather than consequential mechanism in dyslexia. This study contributes to our understanding of the temporal processing deficits underlying the development of dyslexia, but also shows that the development of targeted interventions is still a work in progress.

## Graphical abstract

A phonics‐based and auditory training exerted no impact on neural synchronization in pre‐readers at risk for dyslexia, urging for the development of more successful interventions for reading problems. We did reveal an atypical hemispheric specialization at syllable and phoneme rates in children with dyslexia, even before the onset of reading acquisition. This points towards a possible causal rather than consequential mechanism in dyslexia.

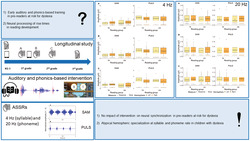

**Abbreviations**
AC: active control
ACNE: Active Control and No Envelope Enhancement intervention group
ADHD: attention deficit hyperactivity disorder
ASD: autism spectrum disorder
ASSRs: Auditory Steady‐State Responses
AST: assymetric sampling in time
CI: confidence interval
COVID‐19: Coronavirus Disease 2019
dBpeSPL: Decibel Peak Equivalent Sound Pressure Level
DC: direct current
DR: children with dyslexia
EE: envelope enhancement
EEG: electroencephalogry
EMM: estimated marginal means
FFT: fast Fourier transform
GGEE: GraphoGame and Envelope Enhancement intervention group
GG‐FL: GraphoGame‐Flemish
GGNE: GraphoGame and No Envelope Enhancement intervention group
Hz: hertz
IQ: intelligence quotient
LIST: Leuven Intelligibility Sentence Test
LK: letter knowledge
M: mean
mV: milivolt
*N*
: number
NE: No Envelope Enhancement
PA: phonological awareness
PULS: pulsatile
RAN: rapid automatized naming
SAM: sinusoidal amplitude modulated
SD: standard deviation
SES: socio‐economic status
TEMPEST: Temporal Envelope Speech Tracking
TR: typically reading children
μV: microvolt

## 1 INTRODUCTION

Literacy plays a significant role in today's culture. Unlike spoken language, the acquisition of written language relies on formal reading instruction (Rastle et al., 2021). Consequently, the majority of children will eventually attain fluent reading skills. However, around 7% of children are eventually diagnosed with developmental dyslexia, meaning that they experience severe and persistent difficulties in reading and/or spelling despite adequate instruction and intelligence and intact sensory abilities (Peterson & Pennington, 2012, 2015).

Targeted reading interventions promote reading and related skills and can support readers with dyslexia. Reading interventions are most successful when they provide explicit and systematic phonics instruction (National Institute of Child Health and Human Development, 2000). Although there is strong empirical support for the efficacy of phonics‐based interventions (Galuschka et al., 2014; Snowling & Hulme, 2011; Wanzek & Vaughn, 2007), in practice, they are often provided at a time that does not allow children with dyslexia to optimally benefit from them (Ozernov‐Palchik & Gaab, 2016). Already in first grade there is an achievement gap between dyslexic and typical readers that persists into adolescence (Ferrer et al., 2015). This calls for early interventions, even as early as kindergarten, with the potential to reduce or even close this gap. Indeed, early reading interventions administered during kindergarten and around the onset of formal literacy education are most effective (Wanzek & Vaughn, 2007). Furthermore, structural brain research demonstrates a natural plasticity window for reading within the first years of primary school and thus further argues for the implementation of early interventions (Van Phan et al., 2021). Nonetheless, interventions are usually only provided after several years of reading instruction and clear reading failure, hence when the most effective time for intervention has already passed. This has been labelled the ‘dyslexia paradox’ (Ozernov‐Palchik & Gaab, 2016).

Phonics‐based interventions, which include systematic and explicit instruction of letter‐sound relations and phoneme blending exercises, have been developed to target the decoding difficulties in dyslexia caused by phonological processing problems (Snowling & Hulme, 2011). This phonological processing deficit is the most widely agreed upon cognitive origin of dyslexia (Snowling, 2000; Vellutino et al., 2004). More recently, phonological difficulties have been associated with an auditory temporal processing deficit, and more specifically an atypical temporal sampling of speech by neural oscillations (Goswami, 2011). The temporal sampling framework (TSF) of Goswami (2011) suggests that neural synchronization of slow‐rate oscillations to the temporal modulations of the speech envelope is atypical in dyslexia. This affects children's phonological development and as a result impedes reading development. Cortical oscillatory frequencies coincide with important amplitude modulation rates in the speech envelope and successful synchronization of neural oscillations to these temporal modulations plays a key role in speech perception and intelligibility (Ghitza & Greenberg, 2009; Giraud & Poeppel, 2012a; Peelle & Davis, 2012). Specifically, synchronization in the delta (<4 Hz) and theta (4–8 Hz) ranges is linked to syllable‐rate processing, whereas synchronization in the beta (13–30 Hz) and gamma (>30 Hz) ranges is associated with phoneme rate processing (Ghitza, 2011; Ghitza & Greenberg, 2009; Poeppel, 2003). Speech perception theories have furthermore proposed hemispheric specialization patterns. The prominent ‘asymmetric sampling in time’ (AST) hypothesis (Poeppel, 2003), for example, assigns right‐hemispheric dominance to syllable rate modulations and a bilateral or left‐hemispheric dominance to phoneme rate modulations (Boemio et al., 2005; Giraud & Poeppel, 2012b; Poeppel, 2003). Considering the importance of neural synchronization for speech segmentation and intelligibility (Doelling et al., 2014; Giraud & Poeppel, 2012a), it has been hypothesized that atypical neural synchronization or hemispheric specialization at these rates in dyslexia could be harmful for speech perception and consequently phonological and reading development (Goswami, 2019).

Over the past years, several studies have investigated the temporal processing deficit in both adults and children with dyslexia (see Lizarazu, Scotto di Covella, et al., 2021, for a review). Studies investigating syllable‐rate processing provided solid evidence for atypical delta synchronization (Keshavarzi et al., 2022; Mandke et al., 2022; Power et al., 2016; Soltész et al., 2013) mostly located in the right hemisphere (Hämäläinen et al., 2012; Lizarazu, Scotto di Covella, et al., 2021; Molinaro et al., 2016). People with dyslexia rather consistently show decreased delta synchronization compared to controls. Results at theta rate are rather inconsistent. Two studies demonstrated decreased theta synchronization in dyslexia (Granados Barbero et al., 2022; Mandke et al., 2022), while two other studies demonstrated increased theta synchronization (Granados Barbero et al., 2021; Lizarazu et al., 2015). In the study of Lizarazu et al. (2015), the dyslexia group also failed to demonstrate a right‐hemispheric lateralization at the theta rate. In the study of Granados Barbero et al. (2022), children with dyslexia demonstrated a belated rightward hemispheric specialization. Using a source analysis methodology, the authors demonstrated a significantly weaker rightward lateralization in children with dyslexia compared to typical readers, Notably, the dyslexic readers caught up with typical readers by the age of 9, pointing towards a late maturation process of hemispheric specialization in dyslexia. Results for phonemic processing in the beta and low‐gamma range are also rather inconclusive. Studies demonstrated decreased synchronization (Granados Barbero et al., 2022; Mandke et al., 2022; Menell et al., 1999) mostly located in the left hemisphere (Lehongre et al., 2011, 2013; Lizarazu, Scotto di Covella, et al., 2021; Poelmans et al., 2012), increased synchronization (De Vos et al., 2017a, 2017b; Granados Barbero et al., 2022), at times restricted to the right hemisphere (Lizarazu et al., 2015), and even typical synchronization (Hämäläinen et al., 2012; Vanvooren et al., 2014). Although not yet fully unraveled, it is hypothesized that people with dyslexia display atypical neural synchronization and differential hemispheric specialization when compared to their typically developing peers. What most studies investigating the temporal processing deficits in dyslexia did not take into account is the importance of amplitude rise times (also called ‘edges’) for sustaining synchronization to the speech signal. Amplitude rise times (from here on rise times) indicate the time it takes to reach peak amplitude in the speech envelope and are most prominent at syllable onsets (Goswami, 2018, 2019). Rise times, similar to amplitude, are one of the measures of the dynamics in the speech envelope. The dynamics in the speech envelope are characterized by the modulation depth at the modulation frequency and rise times of amplitude variations in the envelope. Rise times facilitate temporal sampling of speech by triggering a phase reset of neural oscillations, causing them to synchronize to the temporal modulations present in the speech signal (Gross et al., 2013). It is specifically the sharpness of the edges or rise times that facilitates synchronization (Doelling et al., 2014).

At the behavioral level, difficulties with rise time discrimination and its link with reading development are commonly demonstrated in children (Goswami et al., 2002, 2011; Law et al., 2017; Poelmans et al., 2011; Richardson et al., 2004; Vanvooren et al., 2017) and adults (Hämäläinen et al., 2005; Law et al., 2014; Leong et al., 2011) with dyslexia. However, to the best of our knowledge only two studies have investigated the impact of rise times on neural synchronization in dyslexia (Lizarazu, Lallier, et al., 2021; Van Hirtum, Ghesquière, & Wouters, 2019). Lizarazu, Lallier, et al. (2021) demonstrated that whereas in typical readers, rise times in the speech signal phase reset slow‐rate oscillations (delta and theta), thereby enhancing their synchronization to speech, this mechanism was impaired in dyslexia. The study by Van Hirtum, Ghesquière, and Wouters (2019) showed a deficit in neural synchronization at alpha and beta rate in dyslexia only when the rise times of the stimuli were shortened. This study used Auditory Steady‐State Responses (ASSRs) as a tool to measure neural synchronization (Picton et al., 2003). The studies of Lizarazu, Lallier, et al. (2021) and Van Hirtum, Ghesquière, and Wouters (2019) provide evidence for the impact of rise times on neural synchronization in dyslexia, although studies on the subject are rather scarce and evidence is currently limited to cross‐sectional studies in adults. Investigating the impact of rise times on temporal processing in children before the onset of reading acquisition might allow us to establish whether this is a symptom or a possible cause of dyslexia. A recent study by Van Herck, Economou, et al. (2022) investigated the stimuli of Van Hirtum, Ghesquière, and Wouters (2019) in children and demonstrated that stimuli with shortened rise times elicit much stronger ASSR than regular sinusoidal amplitude modulated stimuli at syllable rate, thereby enhancing their sensitivity. Hence, the authors postulated that using stimuli with shortened rise times might be preferable in developmental reading research to examine the neural mechanisms behind dyslexia. Furthermore, in typically reading children the stimuli with shortened rise times elicited a leftward lateralization at syllable rate, in contrast to the rightward lateralization suggested by AST (Poeppel, 2003). This was proposed to be evoked by the much stronger temporal information in these stimuli, in line with the theory by Zatorre and Belin (2001) that assigned temporal information processing to the left hemisphere. Therefore, using stimuli with shortened rise times might also help unravel the mechanisms behind atypical hemispheric specialization in dyslexia.

Aforementioned studies investigating the temporal processing deficit in dyslexia have improved our understanding of the specific mechanisms involved in the development of dyslexia, and furthermore contribute to the development of more targeted interventions specifically addressing the mechanisms at play. Such targeted interventions addressing the temporal processing deficit for example train auditory temporal processing and rhythm skills and have been shown to improve phonological and literacy development (Bhide et al., 2013; Cancer et al., 2021; Cancer & Antonietti, 2022; Degé & Schwarzer, 2011; Flaugnacco et al., 2015; Harrison et al., 2018). Another promising auditory intervention is the envelope enhancement (EE) intervention of Van Herck, Vanden Bempt, et al. (2022), Vanden Bempt, Economou, et al. (2022) and Vanden Bempt, Van Herck, et al. (2022) in which children listen to natural speech of which the speech envelope is enhanced. EE was originally developed for use in cochlear implant users (Geurts & Wouters, 1999; Koning & Wouters, 2012, 2016) and specifically accentuates rise times in the speech envelope, which facilitates their detection. EE has already been shown to improve speech perception in a speech in noise task passively and instantaneously in both children and adults with dyslexia (Van Hirtum et al., 2021; Van Hirtum, Moncada‐Torres, et al., 2019). Van Herck, Vanden Bempt, et al. (2022) investigated the impact of EE combined with phonics‐based instruction as an intervention for pre‐readers with an elevated cognitive risk of developing dyslexia in a behavioral rise time discrimination task. Notably, listening to stories that were envelope enhanced during an intervention in kindergarten, improved children's rise time sensitivity by the start of formal reading instruction, while the groups that listened to non‐enhanced stories demonstrated improved rise time sensitivity only after 1 year of formal reading instruction in first grade (Van Herck, Vanden Bempt, et al., 2022). The authors concluded that the EE intervention improved rise time discrimination at a crucial time in development, namely prior to the onset of reading instruction and during the most sensitive period for intervention (Ozernov‐Palchik & Gaab, 2016; Van Phan et al., 2021) and that this might give children at risk for dyslexia a head start at the start of reading acquisition. Despite its impact on rise time sensitivity (Van Herck, Vanden Bempt, et al., 2022), the EE intervention did not promote speech perception, phonological awareness or productive letter knowledge (Vanden Bempt, Van Herck, et al., 2022). However, the neural aspects of the EE intervention remain to be investigated.

Despite these recent advances in our understanding of the temporal processing deficit and the development of more targeted interventions for the mechanisms at play, there are still some unresolved questions. First, to our knowledge, no study has yet investigated whether interventions can improve neural synchronization in dyslexia. Providing interventions would be particularly valuable early on in development (Ozernov‐Palchik & Gaab, 2016; Van Phan et al., 2021). Not only is there a need to examine the impact of the frequently administered phonics‐based interventions on neural synchronization but also of an auditory training such as the EE intervention. EE specifically addresses the temporal processing deficit by accentuating rise times in the speech envelope and thereby supposedly enhancing suboptimal neural synchronization (Van Hirtum, Moncada‐Torres, et al., 2019). EE has already been demonstrated to improve behavioral rise time discrimination (Van Herck, Vanden Bempt, et al., 2022), but its efficacy remains to be investigated at the neural level. Second, despite the solid evidence for a deficit in the processing of rise times at the behavioral level in children with dyslexia and its link with later phonology and literacy performance (Goswami et al., 2002, 2011; Law et al., 2017; Poelmans et al., 2011; Richardson et al., 2004; Vanvooren et al., 2017), the neural processing of rise times has not yet been investigated in children with dyslexia. Such an investigation would provide even more valuable insights into the temporal processing deficit, especially when integrated in a longitudinal investigation that enables us to exclude that the observed differences are a consequence of the disorder.

To this end we designed a longitudinal study with a twofold objective. Our first aim was to investigate the impact of an early intervention combining a phonics‐based training and EE on neural synchronization in Dutch pre‐readers at cognitive risk for dyslexia (intervention research question). Secondly, we aimed to investigate the role of atypical neural processing of rise times in reading development (reading groups research question). To answer these aims, we longitudinally investigated ASSRs in children with an elevated cognitive risk of developing dyslexia at pre‐reading (5 years old) and beginning reading age (7 years old) and more specifically prior to, immediately following and approximately 1 year after a digital home‐based intervention. Children's reading skills were measured in third grade. We specifically measured ASSRs in response to 4‐Hz (theta; syllable rate) and 20‐Hz (beta; phoneme rate) amplitude modulated stimuli with varying rise times, namely sinusoidal amplitude modulated (SAM) stimuli and pulsatile stimuli with shortened rise times (PULS). ASSRs were measured in (1) children receiving phonics‐based training and an envelope enhanced story‐listening game, (2) children receiving phonics‐based training and a non‐enhanced story‐listening game and (3) children playing an immersive control game that did not directly train literacy skills and the non‐enhanced story‐listening game. This group design allowed us to disentangle the effects of the EE and phonics‐based training and the inclusion of an active control group eliminated possible placebo effects. Apart from the intervention groups, the third grade reading data allowed for retrospective classification of these children in two reading groups, dyslexic and typical readers, for the purpose of the second research aim of the study.

## 2 MATERIALS AND METHODS

### 2.1 Participants

The total sample consisted of 91 children with an elevated cognitive risk of developing dyslexia. All children were native Dutch speakers with bilateral normal hearing, neither history of brain damage or neurological disorders nor (preliminary) diagnosis of autism spectrum disorder (ASD) or attention deficit hyperactivity disorder (ADHD) did not receive any therapy due to language and/or articulatory problems and had already received a total schooling period of at least 20 months. All children scored above a norm score of 75.3 on the Raven's Colored Progressive Matrices (Raven et al., 1984). Furthermore, to be classified as having an elevated cognitive risk for dyslexia, the children needed to score below the 30th percentile on two out of three reading precursors during a large‐scale screening phase (*N* = 1225) organized in the last year of kindergarten in Flanders (Belgium) (Verwimp et al., 2020). These measures were phonological awareness (PA), letter knowledge (LK) and rapid automatized naming (RAN), the strongest cognitive pre‐literacy predictors of dyslexia (Caravolas et al., 2012; Clayton et al., 2020; Ozernov‐Palchik & Gaab, 2016). Since the phonics‐based training predominantly focused on grapheme‐phoneme coupling, children scoring below the 30th percentile on PA and RAN were additionally required to have LK below the 40th percentile. This ensured that children could optimally benefit from the phonics‐based training. A detailed description of the sample and screening tasks and procedure can be found in previous publications (Economou, Van Herck, et al., 2022; Van Herck, Vanden Bempt, et al., 2022; Vanden Bempt et al., 2021; Verwimp et al., 2020).

**FIGURE 1:**
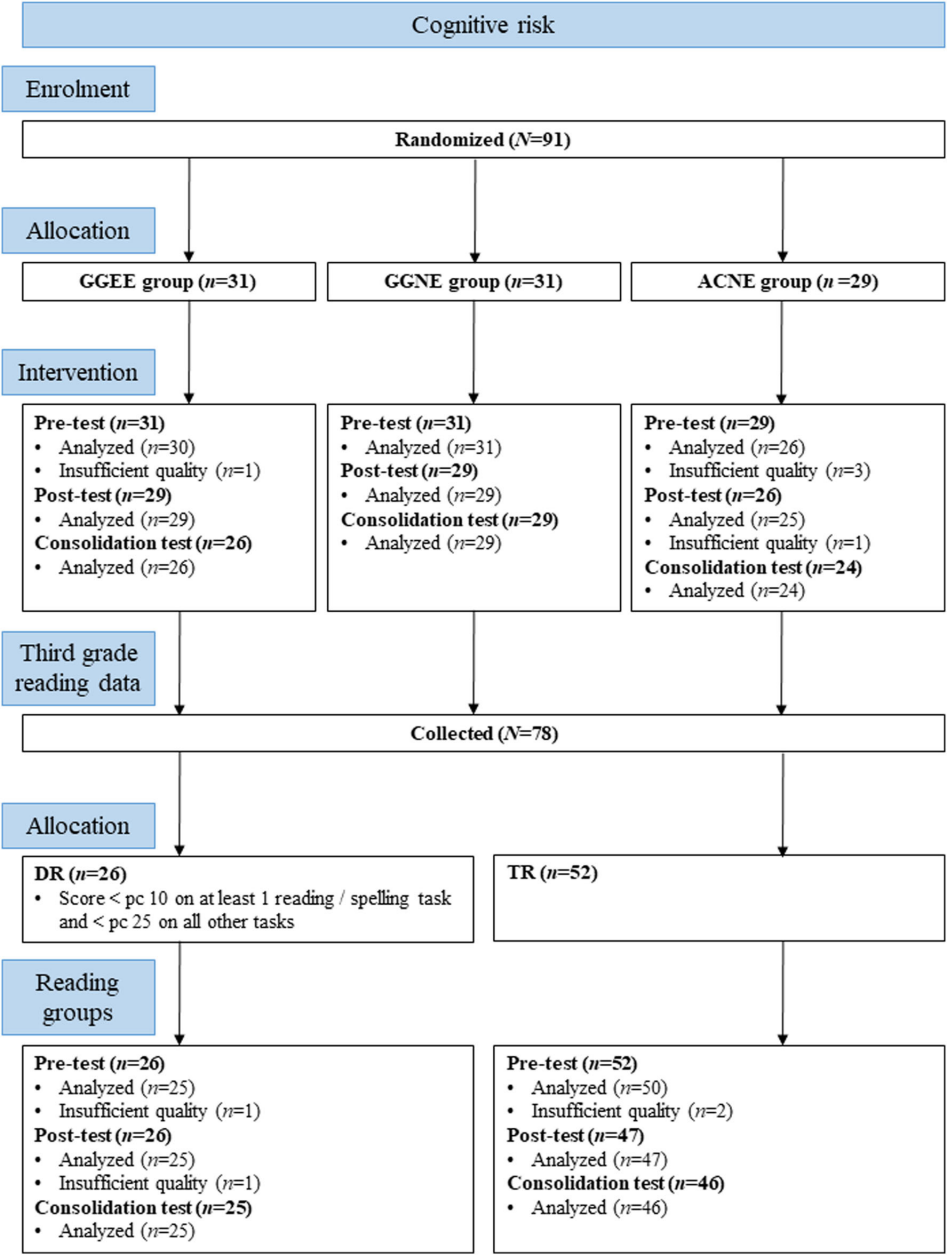
Participant flow in the study

At pre‐test, all 91 children were invited for EEG data collection, during which each child participated in four EEG conditions, resulting in a total of 364 EEG conditions at pre‐test. At post‐test, seven children discontinued participation, resulting in 336 EEG conditions. At consolidation test, two children that did not participate at post‐test returned, while an additional seven children discontinued participation at consolidation test. This resulted in a total of 12 children not participating at consolidation test, and 316 EEG conditions. Furthermore, at each test phase, measurements of some of the EEG conditions were excluded for the following reasons: (1) excessive noise levels, (2) measurement was ended prematurely, (3) data of the specific condition was not collected and (4) uncertainty about the child's hearing of the stimulus. As a result, measurements of 30 out of 364 conditions were excluded at pre‐test, 14 out of 336 at post‐test, and five out of 316 at consolidation test. Note that in case of excluded conditions, available conditions of a child are still included in the analyses. Third grade reading data were collected for 78 children. A detailed description of the participant flow can be found in Figure 1.

For the intervention research question, all 91 children of the total sample were included. We included three experimental groups that each received a slightly different intervention programme: (1) a phonics‐based training and envelope enhanced speech group (GGEE), (2) a phonics‐based training and non‐envelope enhanced speech group (GGNE) and (3) an active control group (ACNE). This was a pseudo‐randomized group assignment at the individual level based on the factors birth trimester, sex and educational environment (i.e., school) and the resulting groups did not differ in age (months), non‐verbal IQ, sex, socio‐economic status (SES) and handedness. The intervention groups had comparable intervention exposure (see Table 1 for demographic data).

**TABLE 1:** Participant characteristics intervention groups

Characteristic	Levels	GGEE (*n* = 31)		GGNE (*n* = 31)		ACNE (*n* = 29)		Group comparison	
		*M*	*SD*	*M*	*SD*	*M*	*SD*	*F*	*p*‐value
Age pre‐test (months)		65	3	65	3	65	4	*F*(2,88) = .27	*p* = .764
Non‐verbal IQ^a^		101	14	101	17	96	13	*F*(2,88) = .98	*p* = .378
Sex	Male/female	19/12		15/16		16/13		*χ* ^2^(2, *N* = 91) = 1.04	*p* = .594
SES^b^	Low/middle/high/missing	8/9/14/0		7/12/12/0		8/15/5/1			*p* = .190
Handedness	Left/ambidexter/right	1/1/29		2/1/28		2/2/25			*p* = .859
Story listening game exposure (hours)		10.5	3.0	11.2	2.8	11.4	1.9	*F*(2,84) = .99	*p* = .370
Phonics‐based training/control game exposure (hours)		16.1	5.3	16.0	5.3	17.1	4.8	*F*(2,88) = .41	*p* = .670

*Note*: GG = GraphoGame; EE = envelope enhanced; NE = not envelope enhanced; AC = active control; *M* = group mean; *SD* = standard deviation; SES = socio‐economic status. For categorical data (i.e., sex, SES and handedness) the numbers in each group are reported instead of *M* and *SD*, and group comparisons are done using Chi‐squared tests (sex) or Fishers's exact tests (SES and handedness) instead of one‐way analysis of variance (ANOVA).

^a^ Reported scores are standardized scores based on the *M* and *SD* of the total group of screened children (*M* = 98.15, *SD* = 16.66).

^b^ SES is based on the parental educational level of the mother.

For the reading groups research question, only the children with available third grade reading data were included, as these data were required for the dyslexia versus typical reading classification. This left us with a sample of 78 children. Based on the acquired reading and spelling data, children were classified as dyslexic if they scored below the 10th percentile on at least one of the included reading or spelling tasks, and below the 25th percentile on all other tasks. All remaining children were classified as typical readers. The tasks included standardized word reading (Brus & Voeten, 1973; Verhoeven, 1995) and pseudoword reading tasks (van den Bos et al., 1994) and a standardized spelling task (Deloof, 2006). This classification resulted in a sample of 26 children with dyslexia (DR) and 52 typically reading children (TR). At pre‐test, the groups did not differ in age (months), non‐verbal IQ, phonological awareness (PA), productive and receptive letter knowledge (LK), sex, SES and handedness. The groups only differed slightly on rapid automatized naming (RAN) (see demographic data in Table 2). Even though the TR children are classified as such based on the available third grade reading data, it is important to keep in mind that they were initially screened in kindergarten for an elevated cognitive risk of developing dyslexia. Therefore, the TR group is not an accurate representation of the general typical reading population, which might provide an explanation for their similar pre‐reading cognitive profile to the DR group.

**TABLE 2:** Participant characteristics reading groups

Characteristic	Levels	TR (*n* = 52)		DR (*n* = 26)		Group comparison	
		M	SD	M	SD	*F*	*p*‐value
Age at pre‐test (months)		65	3	65	3	*F*(1,76) = .02	*p* = .896
Non‐verbal IQ^a^		101	14	96	14	*F*(1,76) = 2.15	*p* = .147
PA^b^		3	3	2	3	*χ* ^2^(1) = 2.57	*p* = .11
RAN^c^		.55	.10	.49	.11	*F*(1,75) = 4.75	*p* < .05
LK productive^d^		2.00	4.00	1.50	2.00	*χ* ^2^(1) = 2.82	*p* = .09
LK receptive^d^		6.50	3.22	5.29	2.46	*F*(1,70) = 2.53	*p* = .12
Sex	Male/female	31/21		14/12		*χ* ^2^(1, *N* = 78) = .06	*p* = .808
SES^e^	Low/middle/high	14/16/22		5/13/8			*p* = .299
Handedness	Left/Ambidexter/right	4/1/47		0/3/23			*p* = .071

*Note*: TR *=* typically reading children; DR = children with dyslexia; *M* = group mean; *SD* = standard deviation; PA = phonological awareness; RAN = rapid automatized naming; LK = letter knowledge; SES = socio‐economic status. For non‐normally distributed data (i.e., phonological awareness and active letter knowledge), the median and interquartile range are presented instead of the mean and standard deviation, and group comparisons are calculated using Kruskal–Wallis rank sum tests instead of one‐way analysis of variance (ANOVA). For categorical data (i.e., sex, SES and handedness) the numbers in each group are reported instead of *M* and *SD*, and group comparisons are done using Chi‐squared tests (sex) or Fishers's exact tests (SES and handedness) instead of one‐way ANOVA.

^a^ Reported scores are standardized scores based on the *M* and *SD* of the total group of screened children (*M* = 98.15, *SD* = 16.66).

^b^ The maximum score for the PA measure was 10.

^c^ RAN raw score was defined as the number of items named correctly per second.

^d^ The maximum score for the LK productive and LK receptive measures was 16.

^e^ SES is based on the parental educational level of the mother.

For both research questions, available data from all children are included in the analyses, since the statistical analyses used in the current study (robust linear mixed‐effects models) are robust to missing data.

The study was approved by the Medical Ethical Committee of the University Hospital of Leuven, KU Leuven, and signed informed consents were obtained for all participants (Katholieke Universiteit Leuven; approval number B322201836276).

### 2.2 Procedure

The data described in the current study were collected as part of a larger longitudinal intervention project (*N* = 149) in which additional behavioral and neuroanatomical assessments were carried out. Additional findings from the intervention study were reported in previous publications (Economou, Van Herck, et al., 2022; Van Herck, Vanden Bempt, et al., 2022; Vanden Bempt et al., 2021). The intervention study comprised a pre‐test, intervention, post‐test, consolidation test and dyslexia status test. Important to note is that the dyslexia status test is not a clinical diagnosis, but rather a classification based on the acquired third grade reading and spelling data. EEG measurements at pre‐test, post‐test and consolidation test were collected in our university research lab, while behavioral assessments (dyslexia status test) were carried out at school in a quiet test room. The intervention took place at home.

In their last year of kindergarten, all children performed an initial baseline measurement of the outcome measure (i.e., ASSRs) at pre‐test. Afterwards, all children performed the tablet‐based intervention at home in which they were instructed to independently play the intervention games for 6 days a week for a total period of 12 weeks. During this intervention, the GGEE and GGNE groups played GraphoGame for 15 min daily in combination with actively listening to stories that were either envelope enhanced (EE; GGEE group) or non‐enhanced (NE; GGNE group) for approximately 10 min. The ACNE group played control games for 15 min and listened to non‐enhanced stories for 10 min. For more details on the practical aspects of the home‐based intervention, see Economou, Van Herck, et al. (2022), Van Herck, Vanden Bempt, et al. (2022) and Vanden Bempt et al. (2021). Post‐test took place shortly after the intervention, at the end of the last year of kindergarten. Consolidation test took place on average 1 year and 2 months after post‐test, after approximately 1 year of formal reading instruction in first grade. In between post‐test and consolidation test, the children received home schooling in first grade, due to the COVID‐19 pandemic. At the start of their third year of primary school, approximately 1 year and 2 months after consolidation test, the children participated in the dyslexia status test.

### 2.3 Interventions

#### 2.3.1 Envelope enhancement

Signal processing of spoken stories was performed in MATLAB R2016b (The MathWorks Inc, 2016). The stimuli and signal processing are identical to those reported in the study by Van Herck, Economou, et al. (2022). Several ways exist to implement a speech processing algorithm such as EE. The one adopted in the current study is only one manner to introduce EE, but has shown to be effective in children and adults with dyslexia (Van Hirtum et al., 2021; Van Hirtum, Moncada‐Torres, et al., 2019). These studies, however, did not use EE as intervention but implemented it in a speech‐in‐noise task during the assessment.

**FIGURE 2:**
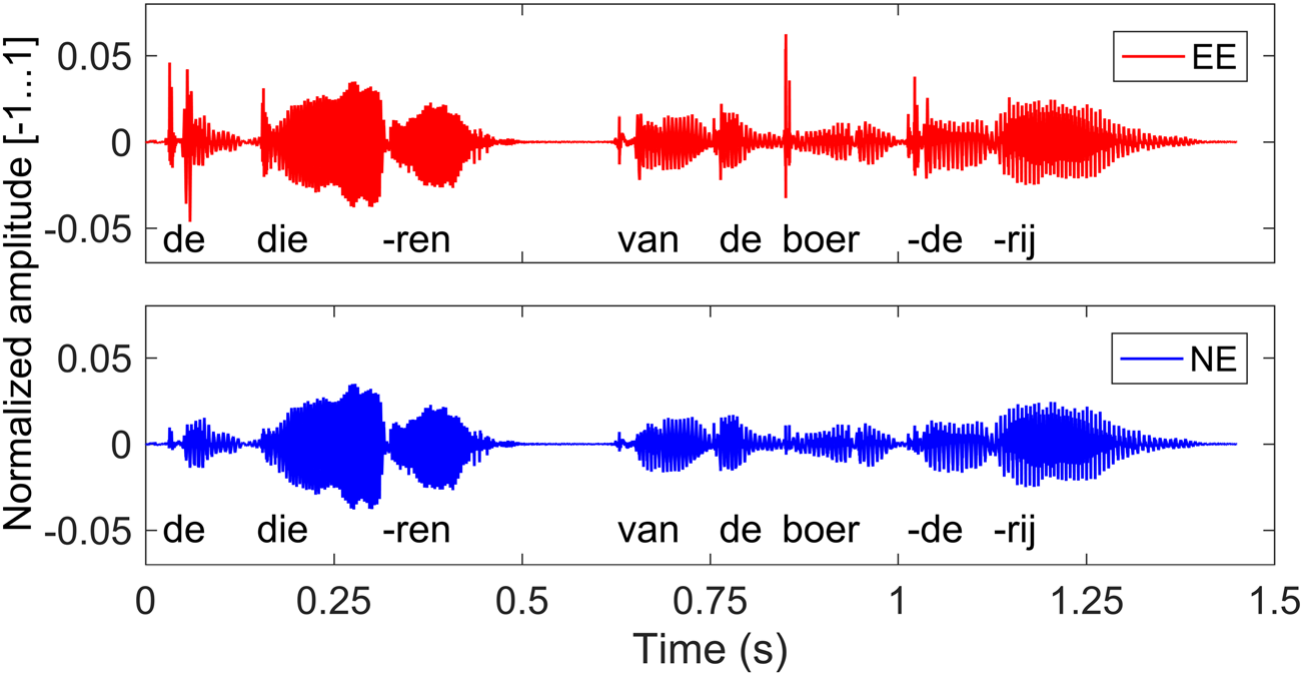
EE/NE stimulus. *Note.* A waveform of a Dutch utterance (English translation: ‘the animals from the farm’) from one of the stories as EE stimulus (upper) and NE stimulus (lower).

As a first step in the signal processing, the original signal $s\left(t\right)$ was resampled to $f_{\text{sampling}}=16\;\mathrm{kHz}$ and subsequently split into frames of 128 samples with a frame advance of 32 samples. A frame‐based fast Fourier transform preceded by Hann windowing transformed the signal to the time‐frequency domain. Upon this, the frequency bins were combined by means of a weighted sum of their powers, mapping them to 23 critical channels. The frequency limits of these channels were defined according to the critical bandwidths of Fastl and Zwicker (2007). The envelope of each channel $E\left(t,k\right)$ was obtained by taking the square root per channel. A slow envelope of each channel $E_{\text{slow}}\left(t,k\right)$ was obtained as follows. The envelope $E\left(t,k\right)$ was low‐pass filtered with a fourth‐order Butterworth filter with a cutoff frequency of 20 Hz, which gave a signal with higher time delay when reacting to sudden increases in $E\left(t,k\right)$. This low‐pass filtered version of $E\left(t,k\right)$ was then half‐wave rectified and amplified by a factor of $A_{\text{slow}}=8$ to obtain $E_{\text{slow}}\left(t,k\right)$. The latter step ensured a higher level of $E_{\text{slow}}\left(t,k\right)$ than that of $E\left(t,k\right)$ at quasi‐stationary parts, while the level of $E\left(t,k\right)$ was higher than that of $E_{\text{slow}\left(t,k\right)}$ at sudden increases in energy (i.e., onsets). As such, subtracting $E_{\text{slow}}\left(t,k\right)$ from $E\left(t,k\right)$ yields a signal with peaks at the onsets of the envelope $E\left(t,k\right)$, and negative values at stationary parts of $E\left(t,k\right)$. A peak envelope signal $E_{\text{peak}}\left(t,k\right)$ could therefore be obtained by subtracting $E_{\text{slow}}\left(t,k\right)$ from $E\left(t,k\right)$, followed by half‐wave rectification and amplification by a factor of $A_{\text{peak}}=3.5$. The eventual peak signal in the time domain $p\left(t\right)$ was obtained by mapping the 23 bands back to 128 frequency bins, followed by an inverse fast Fourier transform after which the frames were recombined by a weighted overlap‐add method. Finally, $p\left(t\right)$ was added to the original signal $s\left(t\right)$, resulting in the envelope enhanced signal $s_{\mathrm{EE}}\left(t\right)$, see Figure 2.

The EE signal processing was applied on age‐appropriate stories that were embedded in a tablet‐based story‐listening game. Two versions of the game were created, one in which the stories were envelope enhanced (EE) and one without enhancement (NE). Regardless of the version of the story‐listening game, the children were instructed to listen to stories for approximately 10 min daily. For more details on the story‐listening game and its feasibility, enjoyment and its impact on language comprehension, see Vanden Bempt, Economou, et al. (2022) and Van Herck, Vanden Bempt, et al. (2022).

#### 2.3.2 GraphoGame

For the purpose of the current study we adapted the GraphoGame interface (Richardson & Lyytinen, 2014) to a tablet‐based Flemish version (GraphoGame‐Flemish, GG‐FL) (Glatz et al., 2021). The training consisted of an introduction of graphemes, visual and auditory discrimination of graphemes and phonemes respectively, and eventually built up to grapheme‐phoneme coupling, phoneme blending and counting, spelling and early reading. During the 12‐week intervention, the children spent 15 min per day on GG‐FL. A detailed description of the training content can be found in Glatz et al. (2021).

#### 2.3.3 Control game

The control game consisted of six commercially available tablet‐based games, namely Lego City My City, LegoDuploTown, LegoDuploTrains, Playmobile horseriding game, Playmobil Police and Lego Heartlake Rush. Children could choose the games they played each day, provided that they played the games for the instructed 15 min per day, identical to GG‐FL.

### 2.4 EEG measures

#### 2.4.1 Recording parameters

EEG signals were recorded with the BioSemi ActiveTwo system using 64 active Ag/AgCL electrodes mounted in head caps according to the 10–20 electrode system. Electrode offsets were kept between −25 and 25 mV. All recordings were administered in a double‐walled soundproof booth with Faraday cage. During the measurement, children were seated in a comfortable chair while watching a soundless movie without subtitles of their choice. This passive listening paradigm ensures a similar level of alertness and attention across subjects throughout the measurement in paediatric ASSR studies (De Vos et al., 2017a; Vanvooren et al., 2014). Measurements were embedded in a different child‐friendly protocol in each test phase. An experienced test leader accompanying the child in the EEG cabin monitored alertness and movement.

For each subject, we collected four EEG conditions (two modulation frequencies × two stimulus types). Each condition lasted approximately 9 min (532.48 s).

#### 2.4.2 Auditory Steady State Responses

**FIGURE 3:**
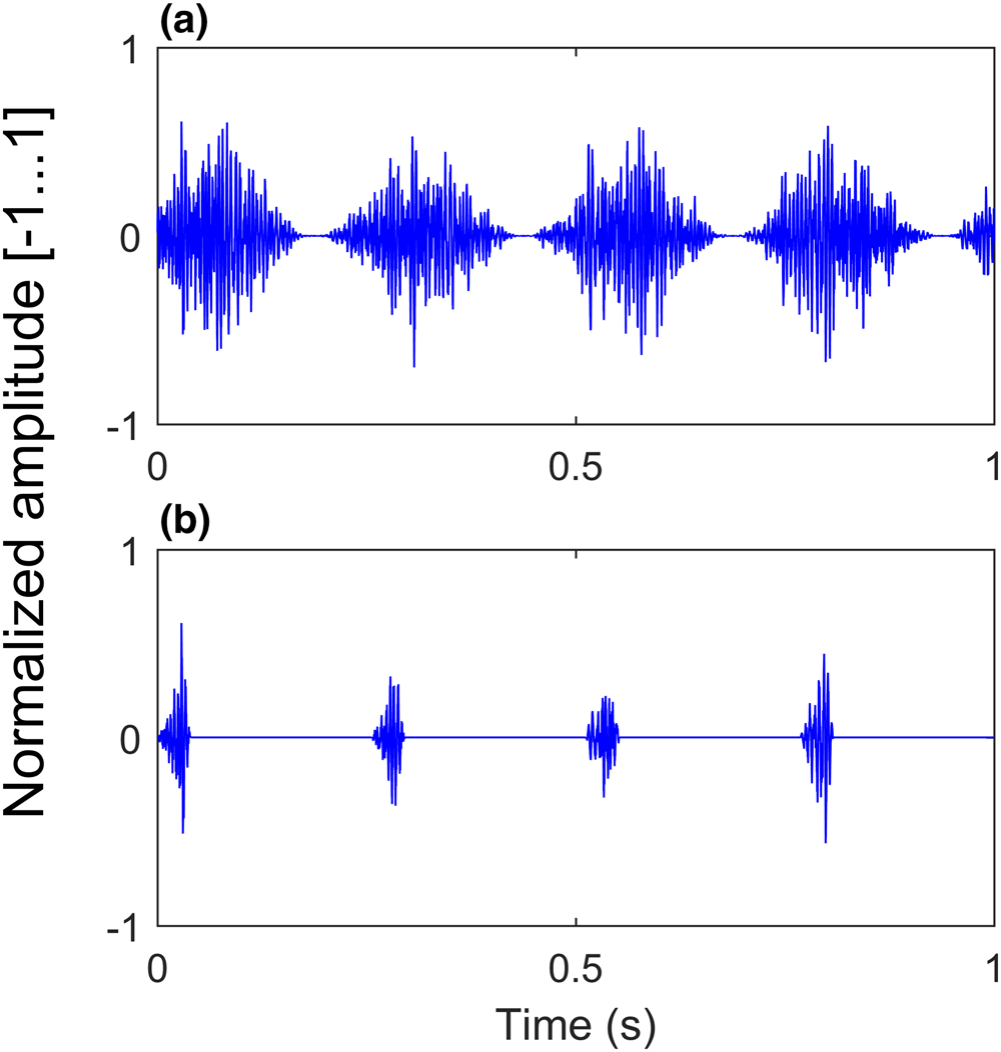
Sinusoidal amplitude modulated (SAM) and pulsatile (PULS) stimulus at 4 Hz. *Note.* A 1‐s waveform of the 4 Hz (a) SAM stimulus and (b) PULS stimulus

ASSRs measure how well the auditory system synchronizes to a stimulus rhythm (Picton et al., 2003). In the current study, we used a speech‐weighted noise as carrier noise to evoke ASSRs. This carrier noise was adopted from the ‘Leuven Intelligibility Sentence Test’ (LIST; Van Wieringen & Wouters, 2008) and represents the long‐term average speech spectrum of 730 sentences of a female speaker. The speech‐weighted noise was 100% amplitude modulated at approximately 4 and 20 Hz to measure neural synchronization of theta and beta oscillations respectively. For each modulation frequency, we created two conditions or stimulus types (see Figure 3): (1) a sinusoidal amplitude modulated condition (SAM) in which a sinusoidal envelope modulation was implemented and (2) a pulsatile condition (PULS) in which the envelope shape was fixed at 30 and 10 ms for rise time and decay time respectively. This resulted in a reduced rise time, without affecting amplitude modulation rates at 4 and 20 Hz. The inclusion of the PULS stimuli allows us to investigate the neural synchronization to rise times.

All stimuli were presented monaurally at 70 dBpeSPL through a calibrated ER‐3B insert earphone to the right ear.

#### 2.4.3 Preprocessing

Preprocessing of the recorded EEG data was done in Matlab R2016b (The MathWorks Inc, 2016). First, we used a high‐pass filter with a second‐order Butterworth filter with a cutoff frequency of 2 Hz to remove the direct current (DC) component. After filtering, the signals were averaged across two pre‐selected electrode configurations, resulting in an artificial left (TP7, P1, P3, P5, P7, P9, PO3, PO7 and O1) and right channel (TP8, P2, P4, P6, P8, P10, PO4, PO8 and O2). This configuration was based on previous research showing that these electrodes are most sensitive to pick up ASSRs in children (Vanvooren et al., 2014). Vanvooren et al. (2014) set a criterion for the sensitivity of an electrode selection so that only electrode pairs with an average amount of significant responses above 70% for all conditions can be included. This criterion was met in the current study, thereby confirming the suitability of the electrode selection in the current dataset. The average number of significant responses for all electrode pairs and all conditions was 87% and 89% for 4 and 20 Hz respectively. Next, the signal was divided into epochs of 1.024 s on which we then applied an epoch‐based artefact rejection to remove muscle potentials and other artefacts. The amplitude rejection level for artefact rejection was set on an individual basis, until 448 epochs remained per electrode, in line with previous ASSR studies in children (De Vos et al., 2017a; Vanvooren et al., 2014, 2015). The aim was to obtain 448 epochs per subject with the lowest amount of artefacts in all channels. Artefact‐free epochs were re‐referenced to electrode Cz. A fast Fourier transform (FFT) algorithm was applied to calculate the complex frequency spectrum for each of the remaining epochs. From the complex frequency spectrum, we obtained the response power, amplitude and phase corresponding to the modulation frequencies used during the experiment (i.e., 4 and 20 Hz, the response spectrum). Mean response amplitudes and phases were computed by vector averaging the complex response spectrum across epochs. The noise amplitude was calculated as the standard deviation of the FFT bin corresponding to the modulation frequency across epochs divided by the square root of the number of epochs. Finally, a one sample Hotelling *T* ^2^ test combining amplitude and phase was performed to determine whether the synchronized activity differed significantly from the neural background activity and hence whether a reliable ASSR (i.e., significant response) was present in the EEG signal (Picton et al., 2003).

### 2.5 Statistical analyses

Statistical analysis was performed in R (version 3.5.1) (R Core Team, 2018). Prior to analysis, Kolmogorov–Smirnov tests were used to assess the assumption of normality, and Levene tests were performed to test for homogeneity of variance. Both assumptions were violated; hence, we adopted robust estimation methods for analysis.

As we did not aim to compare responses across the modulation frequencies, robust linear mixed‐effects models (robustlmm package) (Koller, 2016) were built for each modulation frequency (4 and 20 Hz), outcome measure (response and noise amplitudes) and research question (intervention and reading group) separately. Noise levels determine the precision of the ASSR amplitudes. Therefore, even though there are no hypotheses linked to the noise levels, both response and noise amplitudes are considered in the manuscript. Stimulus type (SAM and PULS), hemisphere (left and right), test phase (pre‐test, post‐test and consolidation test) and intervention group (GGEE, GGNE and ACNE) or reading group (DR and TR) were included in the model as fixed effects and the overarching 4‐way interaction was considered. By‐subject random intercepts accounted for within‐subject observations. The results presented here specifically focus on the effects of intervention and reading groups and possible interaction effects with stimulus type, hemisphere and test phase. The main effects of and interaction effects between stimulus type, hemisphere and test phase will not be discussed since these results apply to all groups and hence do not answer our research questions. These results are the subject of another study by Van Herck, Economou, et al. (2022).

For statistical inference, *p*‐values with a significance level of *p* < .05 were obtained by means of bootstrapping with 1000 simulations for the fixed effects and their interactions in the model. Post hoc comparisons on the interactions were performed using estimated marginal means (EMMs) with a Holm correction for multiple comparison.

## 3 RESULTS

### 3.1 Effects related to the intervention

#### 3.1.1 Response amplitudes

**FIGURE 4:**
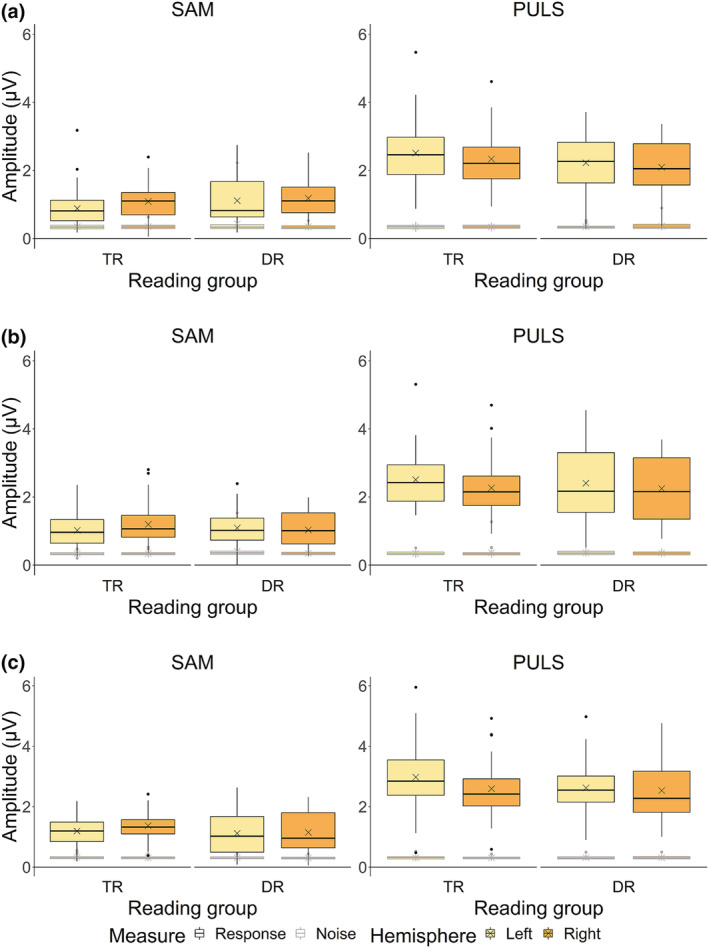
Response and noise amplitudes at 4 Hz for the GraphoGame and Envelope Enhancement intervention (GGEE), GraphoGame and No Envelope Enhancement intervention (GGNE) and Active Control and No Envelope Enhancement intervention (ACNE) groups. *Note.* Panel (a) represents Auditory Steady‐State Responses (ASSRs) raw data in amplitude (μV) for the GGEE group, panel (b) for the GGNE group and panel (c) for the ACNE group. Crosses indicate raw data mean response and noise amplitudes.

For 4‐Hz response amplitudes (Figure 4, boxplots outlined in black), the robust linear mixed‐effects model indicated a significant interaction between intervention group and test phase (*χ* ^2^(4) = 12.54, *p* = .013). Only in the GGNE group there were larger responses at consolidation test compared to pre‐test (*z* = 6.09, *p* < .001, 95% asymptotic CI [.22, .65], Δamplitude = .44 μV) and post‐test (*z* = 3.73, *p* = .003, 95% asymptotic CI [.05, .49], Δamplitude = .27 μV). In none of the other groups did this increase in response reach significance, nor did any of the groups differ from the other at any of the test phases (all *p* > .05). No other effects with intervention group were found (all *p* > .05).

**FIGURE 5:**
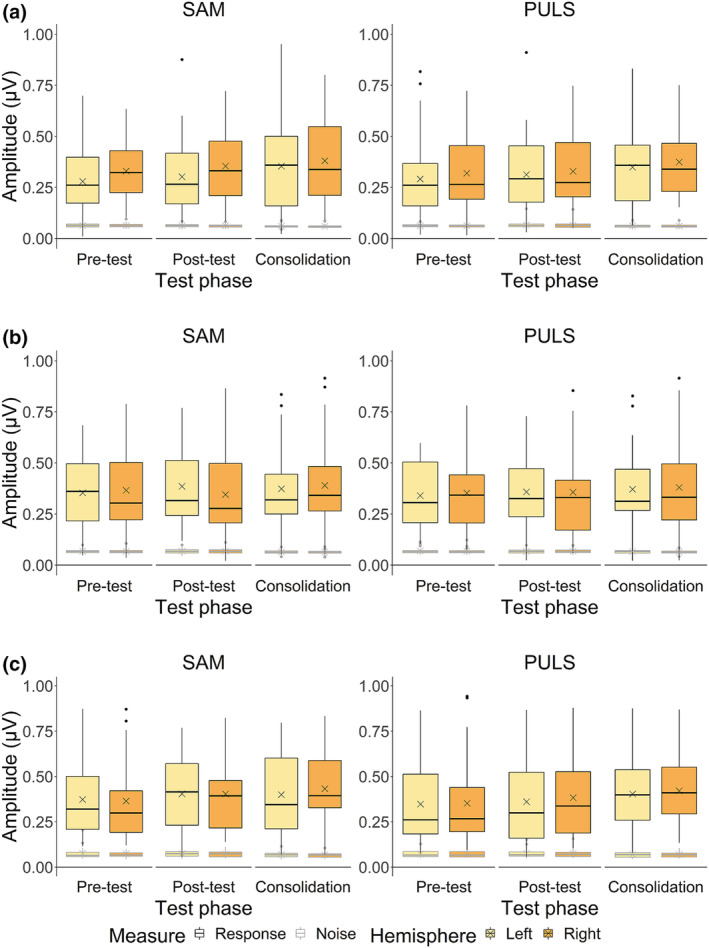
Response and noise amplitudes at 20 Hz for the GraphoGame and Envelope Enhancement intervention (GGEE), GraphoGame and No Envelope Enhancement intervention (GGNE) and Active Control and No Envelope Enhancement intervention (ACNE) groups. *Note.* Panel (a) represents Auditory Steady‐State Responses (ASSRs) raw data in amplitude (μV) for the GGEE group, panel (b) for the GGNE group and panel (c) for the ACNE group. Crosses indicate raw data mean response and noise amplitudes.

At 20 Hz (Figure 5, boxplots outlined in black), no main effect of intervention group, nor any interaction with this factor was found (all *p* > .05).

#### 3.1.2 Noise amplitudes

For 4‐Hz noise amplitudes (Figure 4, boxplots outlined in grey), we found no main effect of intervention group nor any interaction effects with this factor (all *p* > .05).

At 20 Hz (Figure 5, boxplots outlined in grey), a significant main effect of intervention group was found, revealing smaller noise amplitudes in the GGEE compared to the ACNE group only (*z* = −2.69, *p* = .022, 95% asymptotic CI [−.02, −.01], Δamplitude = −.01 μV).

### 3.2 Effects related to dyslexia

Because we were not able to demonstrate intervention effects in the current study, we opted to establish new groups based on third grade reading and spelling data and perform analyses on the resulting reading groups. We fitted models with both intervention group and reading group added as covariate. The interactions between intervention and reading group did not reach significance (all *p* > .05). To further confirm that the intervention had a negligible impact, we subsequently compared two models, one with and one without intervention group as a covariate. Comparison of these models demonstrated no differences between both, so we opted for the simpler model without intervention group included as a covariate for further analyses.

#### 3.2.1 Response amplitudes

**FIGURE 6:**
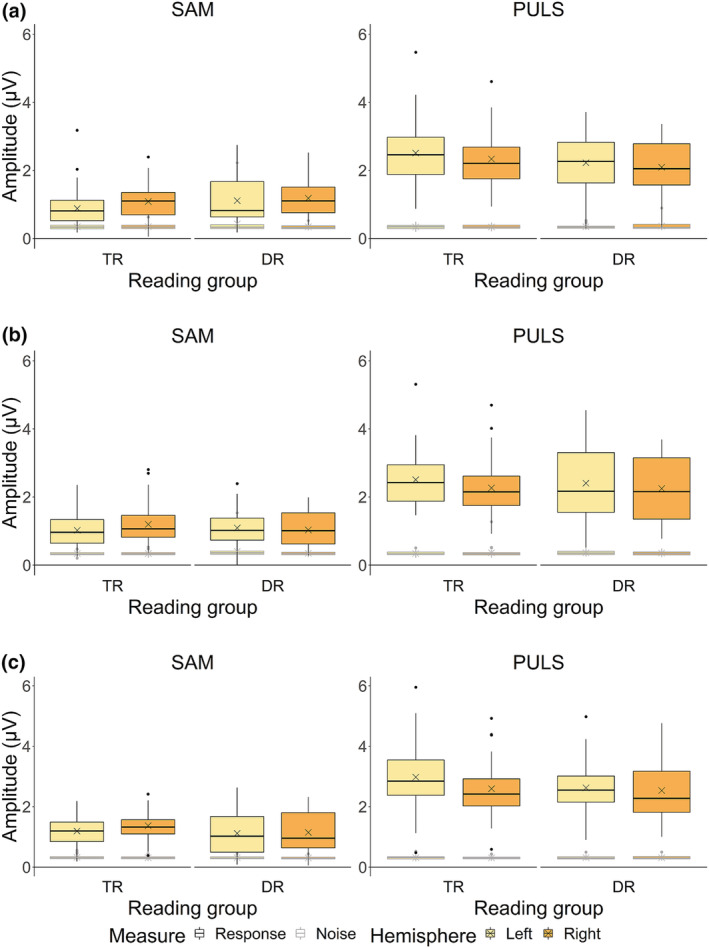
Response and noise amplitudes at 4 Hz for the typically reading children (TR) and children with dyslexia (DR) groups. *Note.* Panel (a) represents Auditory Steady‐State Responses (ASSRs) raw data in amplitude (μV) at pre‐test, panel (b) at post‐test and panel (c) at consolidation test. Crosses indicate raw data mean response and noise amplitudes.

For 4‐Hz response amplitudes (Figure 6, boxplots outlined in black), a significant interaction between reading group, stimulus type and hemisphere was found (*χ* ^2^(1) = 3.79, *p* = .048). This interaction indicated that in typical readers, the SAM stimuli elicited larger responses in the right hemisphere compared to the left hemisphere (*z* = 3.11, *p* = .013, 95% asymptotic CI [.02, .38], Δamplitude = .20 μV), whereas the PULS stimuli elicited larger responses in the left compared to the right hemisphere (*z* = 4.25, *p* < .001, 95% asymptotic CI [.09, .45], Δamplitude = .27 μV). In children with dyslexia however, there was no hemispheric specialization to any of the stimulus types (all *p* > .05). No other effects including the factor reading group were found (all *p* > .05).

**FIGURE 7:**
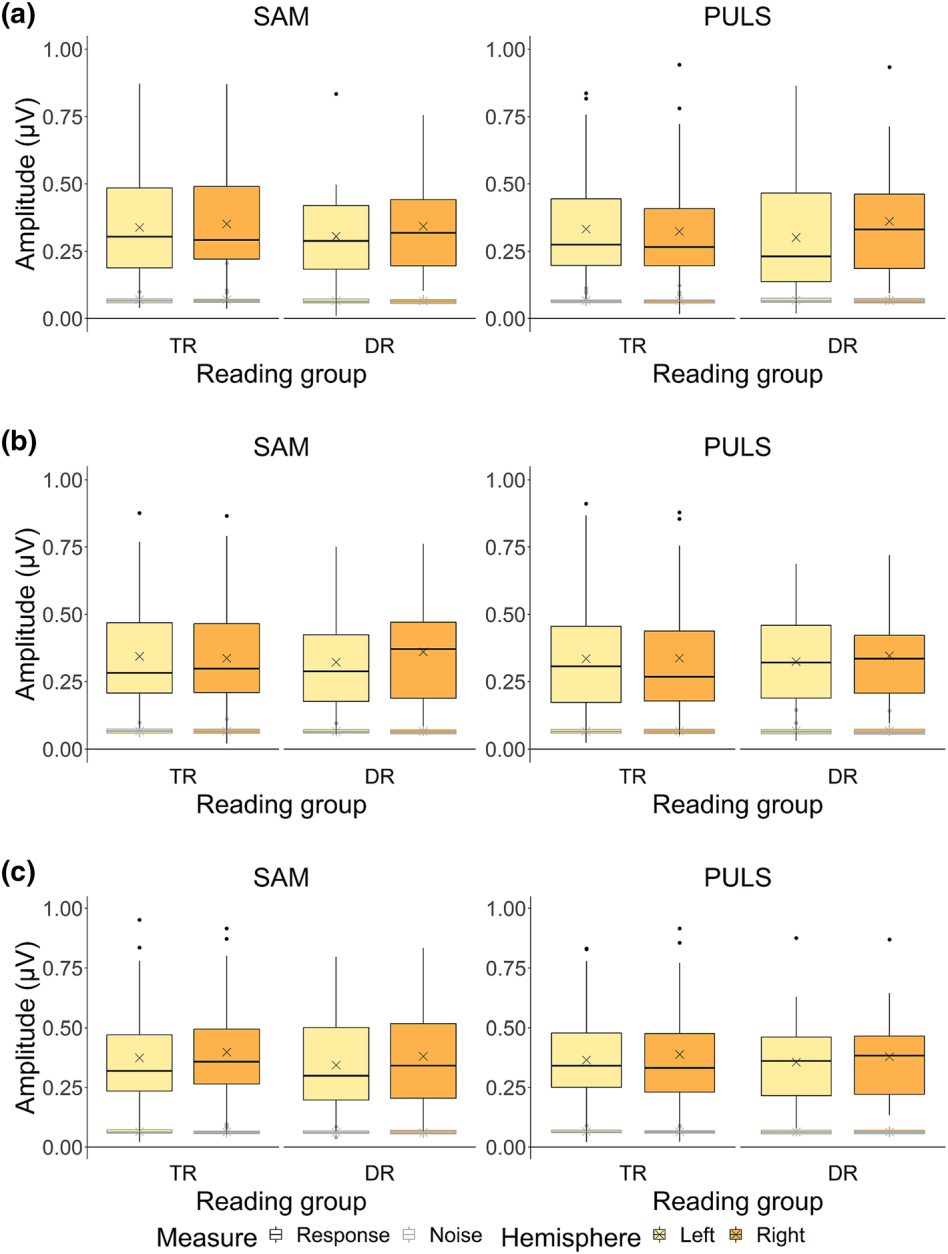
Response and noise amplitudes at 20 Hz for the typically reading children (TR) and children with dyslexia (DR) groups. *Note.* Panel (a) represents Auditory Steady‐State Responses (ASSRs) raw data in amplitude (μV) at pre‐test, panel (b) at post‐test and panel (c) at consolidation test. Crosses indicate raw data mean response and noise amplitudes.

At 20 Hz (Figure 7, boxplots outlined in black), we found a significant interaction between reading group and hemisphere (*χ* ^2^(1) = 4.73, *p* = .027). We confirmed a rightward lateralization in children with dyslexia only (*z* = 3.36, *p* = .003, 95% asymptotic CI [.01, .06], Δamplitude = .04 μV), while there was bilateral processing in typical readers (*p* > .05). No other effects with reading group reached significance (all *p* > .05).

#### 3.2.2 Noise amplitudes

For 4‐Hz noise amplitudes (Figure 6, boxplots outlined in grey) and 20‐Hz noise amplitudes (Figure 7, boxplots outlined in grey), no effects including the factor reading group were found (all *p* > .05).

## 4 DISCUSSION

Dyslexia has been associated with atypical neural synchronization and hemispheric specialization to temporal modulations in the speech envelope (Goswami, 2011; see Lizarazu, Scotto di Covella, et al., 2021, for a review). This is considered to adversely impact speech perception and consequently phonological and reading development (Goswami, 2019). There is a need to investigate the impact of interventions for dyslexia on the temporal processing deficit, as well as to develop new interventions that specifically target the mechanisms at play (i.e., atypical neural synchronization), such as the EE intervention (Van Herck, Vanden Bempt, et al., 2022). Therefore, the first objective of the study was to investigate the impact of an early combined phonics‐based training and EE intervention on neural synchronization in pre‐readers at cognitive risk for dyslexia. Furthermore, amplitude rise times exert an impact on (atypical) neural synchronization in dyslexia (Lizarazu, Lallier, et al., 2021; Van Hirtum, Ghesquière, & Wouters, 2019). Nonetheless, little is known about the causality of the neural processing of rise times in reading development. Accordingly, the second aim of the present study was to longitudinally investigate neural processing of rise times throughout the first years of reading development.

Our results revealed no intervention effects on neural synchronization at syllable and phoneme rate. We did find evidence for an atypical hemispheric specialization at syllable and phoneme rate in children with dyslexia. In typical readers, at syllable rate, the SAM stimuli elicited larger responses in the right compared to the left hemisphere, whereas the PULS stimuli elicited larger responses in the left compared to the right hemisphere. In children with dyslexia, no evidence for any hemispheric specialization was found. Furthermore, at phoneme rate, our results revealed a right hemispheric specialization in children with dyslexia only. These patterns of atypical hemispheric specialization at syllable and phoneme rate were unrelated to the neural processing of rise times.

### 4.1 No intervention effects on neural synchronization to syllable and phoneme rate information

The current study aimed to investigate the impact of an early intervention combining phonics‐based training and an EE intervention, with the latter specifically designed to target temporal processing deficits in dyslexia. The current study could not demonstrate clear intervention effects on neural synchronization at syllable and phoneme rates. Results only showed an increase in syllable rate processing from pre‐test to consolidation test, limited to the GGNE group. This effect, however, was rather small, considering that in none of the test phases the three groups significantly differed from each other. Furthermore, if the effect was truly related to the GraphoGame intervention, we would have expected to uncover the same pattern in the GGEE group, as it is rather unlikely that the EE intervention would have prevented such an increased synchronization from occurring. Therefore, instead of the intervention, we attribute this group difference to random variation within our sample and/or other factors that we might not have explored in the current study. Random individual variability between the GGNE and other groups has already been suggested in the study by Economou, Vanden Bempt, et al. (2022), investigating cortical plasticity following our GraphoGame intervention.

Contrary to our expectations, listening to EE stories did not elicit an intervention effect on neural synchronization to syllable and phoneme rate modulations. We suggest that this is related to the specific mechanisms at play in the temporal processing deficit. While EE was originally suggested to enhance suboptimal neural synchronization to speech (Van Hirtum et al., 2021; Van Hirtum, Moncada‐Torres, et al., 2019), we could not confirm a decreased synchronization to speech‐like stimuli in children with dyslexia in the current study. This might potentially be related to the fact that the typical reading group in the current study was also at cognitive risk for developing dyslexia at pre‐reading age. The underlying deficit we demonstrated seems to entail a more complex pattern of atypical hemispheric specialization (read more about this further on in the discussion), which is unlikely to be resolved by the current EE intervention. Nonetheless, in adults with dyslexia, decreased neural synchronization is demonstrated more regularly (Lehongre et al., 2011, 2013; Lizarazu, Scotto di Covella, et al., 2021; Mandke et al., 2022; Menell et al., 1999; Poelmans et al., 2012; Van Hirtum, Ghesquière, & Wouters, 2019). In this case, EE could possibly normalize reduced neural synchronization. However, we aimed to specifically design an intervention that could be implemented in younger children, considering that this is the most optimal age for interventions and intervening in adults is unlikely to close the gap between typical and dyslexic readers (Ferrer et al., 2015; Ozernov‐Palchik & Gaab, 2016; Van Phan et al., 2021). Hence, even though the current EE intervention might be more effective in adults with dyslexia who do demonstrate reduced neural synchronization, it seems suboptimal for pre‐reading children at risk for dyslexia. This is consistent with the study by Vanden Bempt, Van Herck, et al. (2022) which also failed to demonstrate an EE‐driven effect on speech perception and other reading‐related skills in a largely overlapping population. Speech perception is strongly related to neural synchronization (Ghitza & Greenberg, 2009; Giraud & Poeppel, 2012a; Peelle & Davis, 2012), so the lack of an EE intervention effect on neural synchronization putatively explains the absence of a boosting effect on speech perception and related skills.

Apart from the EE intervention, the current study also investigated the impact of GraphoGame, a frequently implemented phonics‐based training (Economou, Van Herck, et al., 2022; Glatz, 2018; Lovio et al., 2012; Van Herck, Vanden Bempt, et al., 2022; Vanden Bempt et al., 2021). Even though we expected the phonics‐based training to influence phoneme rate processing, considering that formal reading instruction induces increased phoneme rate synchronization (De Vos et al., 2017a), we could not demonstrate an impact of GraphoGame on neural synchronization. However, this finding agrees with the behavioral results in the study of Vanden Bempt et al. (2021). Vanden Bempt et al. (2021) demonstrated that even though playing GraphoGame induced significant short‐term increases in productive and receptive letter knowledge and word decoding, it did not evoke a transfer effect on phonological abilities. Beta oscillations (13–30 Hz) are specifically related to phonetic features or phoneme processing (Ghitza, 2011; Leong & Goswami, 2015) and have been found to be associated with phonological awareness (De Vos et al., 2017a, 2017b; Poelmans et al., 2012). Consequently, it is unsurprising that if GraphoGame did not elicit measurable growth in phonological awareness (Vanden Bempt et al., 2021), there is also no impact on phoneme rate neural synchronization.

Stimulus‐induced noise at phoneme rate (20 Hz) demonstrated a minor, but general difference between the intervention groups. Noise levels were smaller in the GGEE, compared to the ACNE group. It is unclear what might have caused this difference between the groups, as it seems unrelated to aspects of the intervention. Therefore, we suggest that this group difference is another manifestation of the random variation present within our study sample.

### 4.2 Atypical hemispheric specialization at syllable and phoneme rate in children with dyslexia

Our results at syllable rate (4 Hz; theta range) showed that in typical readers the SAM and PULS stimuli elicited a different hemispheric specialization. Whereas responses to SAM stimuli were lateralized to the right hemisphere, responses to PULS stimuli were lateralized to the left hemisphere. Interestingly, responses to both stimulus types were processed bilaterally in children with dyslexia.

While there is strong support for atypical hemispheric specialization at syllable rate when considering the delta range (Hämäläinen et al., 2012; Lizarazu, Scotto di Covella, et al., 2021; Molinaro et al., 2016), evidence in the theta range is far more restricted. Yet, the current study is not the first in revealing an atypical hemispheric specialization in the theta range in dyslexia (Granados Barbero et al., 2022; Lizarazu et al., 2015; Mandke et al., 2022). Consistent with the current results for the SAM stimuli, the studies of Lizarazu et al. (2015) and Mandke et al. (2022) demonstrated a rightward lateralization at syllable rate in typical readers, but a lack thereof in dyslexic readers. A plausible interpretation for this finding can be found in Granados Barbero et al. (2022), who attributed a weaker rightward lateralization in children with dyslexia to a late maturation process that normalized by the age of nine. The children in the current study were only 7–8 years old at the latest test phase, but the data indeed seem consistent with a late maturation for lateralization, which might impact reading development.

We did not only reveal an atypical hemispheric specialization at syllable rate in response to the SAM stimuli, but also in response to the PULS stimuli, which were designed to specifically assess the neural processing of rise times. Whereas the SAM stimuli were lateralized to the right hemisphere, in line with the AST hypothesis (Poeppel, 2003), the PULS stimuli elicited a leftward lateralization in typical readers (Van Herck, Economou, et al., 2022). If a leftward lateralization in response to the PULS stimuli is indeed evoked by stronger temporal information in these stimuli, as hypothesized by Van Herck, Economou, et al. (2022), the lack of such a leftward lateralization in children with dyslexia might imply a reduced sensitivity to the increased temporal information in the PULS stimuli. This would furthermore confirm a deficit in the neural processing of rise times. However, our results rather point to a more global deficit in hemispheric specialization at syllable rate at this age. Children with dyslexia demonstrated an atypical hemispheric specialization in response to both SAM and PULS stimuli, therefore contradicting the claim that the deficit is specifically linked to the processing of shorter rise times in the PULS stimuli.

This is confirmed by our results at phoneme rate (20 Hz), demonstrating a bilateral processing in typical readers, but a rightward lateralization in children with dyslexia. This finding was the same for both the SAM and PULS stimuli, so it seems to be unrelated to the neural processing of rise times. Whereas bilateral processing is considered to aid speech processing (Boemio et al., 2005), multiple studies have failed to demonstrate this in readers with dyslexia and rather demonstrated a right hemispheric preference for processing phoneme rate modulations (Granados Barbero et al., 2022; Lehongre et al., 2011; Lizarazu et al., 2015), consistent with our results. The current study is the first to establish this atypical hemispheric specialization already at pre‐reading age.

Taken together, the current study demonstrated atypical hemispheric specialization at both syllable and phoneme rates of speech in children with dyslexia. Given that both hemispheres are not equally sensitive to syllable and phoneme rate modulations, and hemispheric specialization is therefore important for successful speech processing (Boemio et al., 2005; Giraud & Poeppel, 2012b; Poeppel, 2003), one might suggest that a disruption of this lateralization, as seen in the current study, might affect the efficiency of the auditory processing of speech (Lehongre et al., 2013). This is consistent with the temporal sampling framework of dyslexia, that assigns phonological difficulties, and as a result, reading difficulties, essentially to an atypical temporal sampling of speech (Goswami, 2011).

Importantly, we demonstrated an atypical hemispheric specialization across all test phases included in the current study, ranging from pre‐reading (5 years old) to beginning reading age (7 years old). With this longitudinal design, we aimed to disentangle causal from consequential effects. Considering that the deficits were present already before the onset of reading acquisition, we postulate that an atypical hemispheric specialization might be a potential cause, rather than a consequence of developing dyslexia.

Despite these valuable insights into the deficits underlying the development of dyslexia, we could not confirm the impact of rise times on neural synchronization in children as the studies of Lizarazu, Lallier, et al. (2021) and Van Hirtum, Ghesquière, and Wouters (2019) did in adults. A possibility is that the impact of rise times on neural synchronization only develops at a later age. Another possibility and an important factor to take into account is that our results might be related to the comparison of children with dyslexia to typical readers who had a cognitive risk of developing dyslexia at pre‐reading age. This means that our typical readers are not a true representation of the general population of typical readers and might be outperformed by the latter. We propose that the group differences in the current study might be enhanced when comparing children with dyslexia to true typical readers, or that group differences that we failed to reveal might emerge. Nonetheless, the current study provides an important glance on the deficits underlying the development of dyslexia.

### 4.3 Limitations

A few limitations should be noted in the current study. A first limitation concerns the reading groups comparison. All children included in the analyses for this research question received some kind of intervention in their last year of kindergarten and are thus not typical children with dyslexia or typical readers. Since we could not demonstrate intervention effects and statistical analyses showed no differences between a model with and without intervention group as a covariate, we omitted the intervention group factor in subsequent reading group analyses. However, absence of evidence is not evidence of absence. Ideally, we would have compared children that did not (yet) receive some kind of intervention, which could be addressed in future research.

Inherent to measuring ASSRs is that we are only able to measure one specific modulation frequency at a time. We defined syllable and phoneme rate modulations as 4‐ and 20‐Hz modulations respectively. However, a growing number of studies point towards deficits in the delta instead of theta range for syllable rate processing (Hämäläinen et al., 2012; Lizarazu, Scotto di Covella, et al., 2021; Molinaro et al., 2016; Power et al., 2016; Soltész et al., 2013) and in the gamma instead of beta range for phoneme rate processing (Lehongre et al., 2011, 2013; Lizarazu et al., 2015; Lizarazu, Scotto di Covella, et al., 2021). We suggest future research to therefore include delta rate measurements, for example, at 2 Hz, and gamma rate measurements, for example, at 30 Hz. Alternatively, these issues can be addressed by implementing the recently developed Temporal Envelope Speech Tracking or TEMPEST stimulus framework (Gransier & Wouters, 2021), which allows the evaluation of a range of envelope modulations concurrently within one stimulus.

## 5 CONCLUSION

In conclusion, the current study investigated (1) the impact of an early intervention combining phonics‐based and EE training in pre‐reading children at cognitive risk for developing dyslexia and (2) the role of atypical neural processing of rise times in reading development. Performing a phonics‐based and/or EE training had no impact on neural synchronization at syllable and phoneme rate, thereby urging for the development of interventions that are more successful in remediating the temporal processing difficulties related to the development of dyslexia. Whereas we could not demonstrate a deficit in the neural processing of rise times, our results did reveal a more global atypical hemispheric specialization at both syllable and phoneme rates, even before the onset of formal reading acquisition. Overall, the current study contributes to our understanding of the specific temporal processing mechanisms involved in the development of dyslexia but also exposes that the development of targeted interventions is still a work in progress.

## CONFLICT OF INTEREST

The authors declare no competing interests.

## AUTHOR CONTRIBUTIONS

**Shauni Van Herck:** Formal analysis, investigation, data curation, writing–original draft, visualization and project administration. **Maria Economou:** Writing–Review and editing and project administration. **Femke Vanden Bempt:** Writing–review and editing and project administration. **Toivo Glatz**: Investigation, writing–review and editing and software. **Pol Ghesquière:** Conceptualization, methodology, writing–review and editing, supervision and funding acquisition. **Maaike Vandermosten:** Conceptualization, methodology, writing–review and editing, supervision and funding acquisition. **Jan Wouters:** Conceptualization, methodology, writing–review and editing, supervision and funding acquisition.

### PEER REVIEW

The peer review history for this article is available at https://publons.com/publon/10.1111/ejn.15894.

## Data Availability

The data that support the findings of this study are available on request from the corresponding author. The data are not publicly available due to privacy or ethical restrictions.

## References

[ejn15894-bib-0001] Bhide, A. , Power, A. , & Goswami, U. (2013). A rhythmic musical intervention for poor readers: A comparison of efficacy with a letter‐based intervention. Mind, Brain, and Education, 7(2), 113–123. 10.1111/mbe.12016

[ejn15894-bib-0002] Boemio, A. , Fromm, S. , Braun, A. , & Poeppel, D. (2005). Hierarchical and asymmetric temporal sensitivity in human auditory cortices. Nature Neuroscience, 8(3), 389–395. 10.1038/nn1409 15723061

[ejn15894-bib-0003] Brus, B. T. , & Voeten, M. J. M. (1973). Een Minuut Test, vorm A en B. Verantwoording en handleiding.

[ejn15894-bib-0004] Cancer, A. , & Antonietti, A. (2022). Music‐based and auditory‐based interventions for Reading difficulties: A literature review. Heliyon, 8(April), e09293. 10.1016/j.heliyon.2022.e09293 35497042 PMC9048091

[ejn15894-bib-0005] Cancer, A. , Sarti, D. , De Salvatore, M. , Granocchio, E. , Pia, D. , Chieffo, R. , & Antonietti, A. (2021). Dyslexia Telerehabilitation during the COVID‐19 pandemic: Results of a rhythm‐based intervention for Reading. Children, 8(11), 1011. 34828724 10.3390/children8111011 PMC8624373

[ejn15894-bib-0006] Caravolas, M. , Lervåg, A. , Mousikou, P. , Efrim, C. , Litavský, M. , Onochie‐Quintanilla, E. , Salas, N. , Schöffelová, M. , Defior, S. , Mikulajová, M. , Seidlová‐Málková, G. , & Hulme, C. (2012). Common patterns of prediction of literacy development in different alphabetic orthographies. Psychological Science, 23(6), 678–686. 10.1177/0956797611434536 22555967 PMC3724272

[ejn15894-bib-0007] Clayton, F. J. , West, G. , Sears, C. , Hulme, C. , & Lervåg, A. (2020). A longitudinal study of early Reading development: Letter‐sound knowledge, phoneme awareness and RAN, but not letter‐sound integration, predict variations in Reading development. Scientific Studies of Reading, 24(2), 91–107. 10.1080/10888438.2019.1622546

[ejn15894-bib-0008] De Vos, A. , Vanvooren, S. , Vanderauwera, J. , Ghesquière, P. , & Wouters, J. (2017a). A longitudinal study investigating neural processing of speech envelope modulation rates in children with (a family risk for) dyslexia. Cortex, 93, 206–219. 10.1016/j.cortex.2017.05.007 28686908

[ejn15894-bib-0009] De Vos, A. , Vanvooren, S. , Vanderauwera, J. , Ghesquière, P. , & Wouters, J. (2017b). Atypical neural synchronization to speech envelope modulations in dyslexia. Brain and Language, 164, 106–117. 10.1016/j.bandl.2016.10.002 27833037

[ejn15894-bib-0010] Degé, F. , & Schwarzer, G. (2011). The effect of a music program on phonological awareness in preschoolers. Frontiers in Psychology, 2, 1–7. 10.3389/fpsyg.2011.00124 21734895 PMC3121007

[ejn15894-bib-0011] Deloof, G. (2006). Leerlingenvolgsysteem LVS‐VCLB Spelling 1–2–3‐4‐5‐6. Garant.

[ejn15894-bib-0012] Doelling, K. , Arnal, L. , Ghitza, O. , & Poeppel, D. (2014). Acoustic landmarks drive delta‐theta oscillations to enable speech comprehension by facilitating perceptual parsing. NeuroImage, 85(2), 761–768. 10.1016/j.neuroimage.2013.06.035.Acoustic 23791839 PMC3839250

[ejn15894-bib-0013] Economou, M. , Van Herck, S. , Vanden Bempt, F. , Glatz, T. , Wouters, J. , Ghesquière, P. , Vanderauwera, J. , & Vandermosten, M. (2022). Investigating the impact of early literacy training on White matter structure in prereaders at risk for dyslexia. Cerebral Cortex, 32, 4684–4697. 10.1093/cercor/bhab510 35059709

[ejn15894-bib-0014] Economou, M. , Vanden Bempt, F. , Van Herck, S. , Wouters, J. , Ghesquière, P. , Vanderauwera, J. , & Vandermosten, M. (2022). Cortical structure in pre‐readers at cognitive risk for dyslexia: baseline differences and response to intervention. Manuscript in Preparation. 10.1162/nol_a_00122 PMC11093402 38832361

[ejn15894-bib-0092] Fastl, H. , & Zwicker, E. (2007). Information processing in the auditory system. In Psychoacoustics: Facts and models (pp. 23–60). Springer Berlin Heidelberg. 10.1007/978-3-540-68888-4_3

[ejn15894-bib-0015] Ferrer, E. , Shaywitz, B. A. , Holahan, J. M. , Marchione, K. E. , Michaels, R. , & Shaywitz, S. E. (2015). Achievement gap in Reading is present as early as first grade and persists through adolescence. Journal of Pediatrics, 167(5), 1121–1125.e2. 10.1016/j.jpeds.2015.07.045 26323201

[ejn15894-bib-0016] Flaugnacco, E. , Lopez, L. , Terribili, C. , Montico, M. , Zoia, S. , & Schön, D. (2015). Music training increases phonological awareness and reading skills in developmental dyslexia: A randomized control trial. PLoS ONE, 10(9), 1–17. 10.1371/journal.pone.0138715 PMC4583182 26407242

[ejn15894-bib-0017] Galuschka, K. , Ise, E. , Krick, K. , & Schulte‐Körne, G. (2014). Effectiveness of treatment approaches for children and adolescents with reading disabilities: A meta‐analysis of randomized controlled trials. PLoS ONE, 9(2), e89900. 10.1371/journal.pone.0089900 24587110 PMC3935956

[ejn15894-bib-0018] Geurts, L. , & Wouters, J. (1999). Enhancing the speech envelope of continuous interleaved sampling processors for cochlear implants. The Journal of the Acoustical Society of America, 105(4), 2476–2484. 10.1121/1.426851 10212428

[ejn15894-bib-0019] Ghitza, O. (2011). Linking speech perception and neurophysiology: Speech decoding guided by cascaded oscillators locked to the input rhythm. Frontiers in Psychology, 2(JUN), 1, 130–13. 10.3389/fpsyg.2011.00130 21743809 PMC3127251

[ejn15894-bib-0020] Ghitza, O. , & Greenberg, S. (2009). On the possible role of brain rhythms in speech perception: Intelligibility of time‐compressed speech with periodic and aperiodic insertions of silence. Phonetica, 66(1–2), 113–126. 10.1159/000208934 19390234

[ejn15894-bib-0021] Giraud, A. , & Poeppel, D. (2012b). Speech perception from a neurophysiological perspective. In The human auditory cortex (Vol. 43). Springer. 10.1007/978-1-4614-2314-0

[ejn15894-bib-0022] Giraud, A.‐L. , & Poeppel, D. (2012a). Cortical oscillations and speech processing: Emerging computational principles and operations. Nature Neuroscience, 15(4), 511–517. 10.1038/nbt.3121.ChIP-nexus 22426255 PMC4461038

[ejn15894-bib-0023] Glatz, T. (2018). Serious games as a level playing field for early literacy: A behavioural and neurophysiological evaluation. [Doctoral Dissertation]. University of Groningen.

[ejn15894-bib-0024] Glatz, T. , Vanderauwera, J. , Vanden Bempt, F. , Economou, M. , Wouters, J. , Vandermosten, M. , & Ghesquiére, P. (2021). Design and structure of GraphoGame‐Flemish, an app‐based tool to support early reading acquisition. OSF. 10.17605/OSF.IO/CYKED

[ejn15894-bib-0025] Goswami, U. (2011). A temporal sampling framework for developmental dyslexia. Trends in Cognitive Sciences, 15(1), 3–10. 10.1016/j.tics.2010.10.001 21093350

[ejn15894-bib-0026] Goswami, U. , Fosker, T. , Huss, M. , Mead, N. , & SzuCs, D. (2011). Rise time and formant transition duration in the discrimination of speech sounds: The Ba‐Wa distinction in developmental dyslexia. Developmental Science, 14(1), 34–43. 10.1111/j.1467-7687.2010.00955.x 21159086

[ejn15894-bib-0027] Goswami, U. , Thomson, J. , Richardson, U. , Stainthorp, R. , Hughes, D. , Rosen, S. , & Scott, S. K. (2002). Amplitude envelope onsets and developmental dyslexia: A new hypothesis. Proceedings of the National Academy of Sciences, 99(16), 10911–10916. 10.1073/pnas.122368599 PMC125072 12142463

[ejn15894-bib-0028] Goswami, U. (2018). A neural basis for phonological awareness? An oscillatory temporal‐sampling perspective. Current Directions in Psychological Science, 27(1), 56–63. 10.1177/0963721417727520

[ejn15894-bib-0029] Goswami, U. (2019). A neural oscillations perspective on phonological development and phonological processing in developmental dyslexia. Lang & Ling Compass, 13(5), 1–21. 10.1111/lnc3.12328

[ejn15894-bib-0030] Granados Barbero, R. , de Vos, A. , Ghesquière, P. , & Wouters, J. (2021). Atypical processing in neural source analysis of speech envelope modulations in adolescents with dyslexia. European Journal of Neuroscience, 54(11), 7839–7859. 10.1111/ejn.15515 34730259

[ejn15894-bib-0031] Granados Barbero, R. , Ghesquière, P. , & Wouters, J. (2022). Development of atypical reading at ages 5 to 9 years and processing of speech envelope modulations in the brain. Frontiers in Computational Neuroscience, 16, 1–26. 10.3389/fncom.2022.894578 PMC9248325 35782088

[ejn15894-bib-0032] Gransier, R. , & Wouters, J. (2021). Neural auditory processing of parameterized speech envelopes. Hearing Research, 412, 108374. 10.1016/j.heares.2021.108374 34800800

[ejn15894-bib-0033] Gross, J. , Hoogenboom, N. , Thut, G. , Schyns, P. , Panzeri, S. , Belin, P. , & Garrod, S. (2013). Speech rhythms and multiplexed oscillatory sensory coding in the human brain. PLoS Biology, 11(12), e1001752. 10.1371/journal.pbio.1001752 24391472 PMC3876971

[ejn15894-bib-0034] Hämäläinen, J. A. , Leppänen, P. H. T. , Torppa, M. , Müller, K. , & Lyytinen, H. (2005). Detection of sound rise time by adults with dyslexia. Brain and Language, 94, 32–42. 10.1016/j.bandl.2004.11.005 15896381

[ejn15894-bib-0035] Hämäläinen, J. A. , Rupp, A. , Soltész, F. , Szücs, D. , & Goswami, U. (2012). Reduced phase locking to slow amplitude modulation in adults with dyslexia: An MEG study. NeuroImage, 59(3), 2952–2961. 10.1016/j.neuroimage.2011.09.075 22001790

[ejn15894-bib-0036] Harrison, E. , Wood, C. , Holliman, A. J. , & Vousden, J. I. (2018). The immediate and longer‐term effectiveness of a speech‐rhythm‐based reading intervention for beginning readers. Journal of Research in Reading, 41(1), 220–241. 10.1111/1467-9817.12126

[ejn15894-bib-0037] Keshavarzi, M. , Mandke, K. , Macfarlane, A. , Parvez, L. , Gabrielczyk, F. , Wilson, A. , & Goswami, U. (2022). Atypical Delta‐band phase consistency and atypical preferred phase in children with dyslexia during neural entrainment to rhythmic audio‐visual speech. NeuroImage: Clinical, 35, 103054. 10.2139/ssrn.3982171 35642984 PMC9136320

[ejn15894-bib-0038] Koller, M. (2016). Robustlmm: An R package for robust estimation of linear mixed‐effects models. Journal of Statistical Software, 75(1), 1–24. 10.18637/jss.v075.i06 32655332 PMC7351245

[ejn15894-bib-0039] Koning, R. , & Wouters, J. (2012). The potential of onset enhancement for increased speech intelligibility in auditory prostheses. The Journal of the Acoustical Society of America, 132(4), 2569–2581. 10.1121/1.4748965 23039450

[ejn15894-bib-0040] Koning, R. , & Wouters, J. (2016). Speech onset enhancement improves intelligibility in adverse listening conditions for cochlear implant users. Hearing Research, 342, 13–22. 10.1016/j.heares.2016.09.002 27697583

[ejn15894-bib-0041] Law, J. M. , Vandermosten, M. , Ghesquiere, P. , & Wouters, J. (2014). The relationship of phonological ability, speech perception, and auditory perception in adults with dyslexia. Frontiers in Human Neuroscience, 8, 1–12. 10.3389/fnhum.2014.00482 25071512 PMC4078926

[ejn15894-bib-0042] Law, J. M. , Wouters, J. , & Ghesquière, P. (2017). The influences and outcomes of phonological awareness: A study of MA, PA and auditory processing in pre‐readers with a family risk of dyslexia. Developmental Science, 20, e12453. 10.1111/desc.12453 27774757

[ejn15894-bib-0043] Lehongre, K. , Morillon, B. , Giraud, A. L. , & Ramus, F. (2013). Impaired auditory sampling in dyslexia: Further evidence from combined fMRI and EEG. Frontiers in Human Neuroscience, 7(JUL), 1–8. 10.3389/fnhum.2013.00454 23950742 PMC3738857

[ejn15894-bib-0044] Lehongre, K. , Ramus, F. , Villiermet, N. , Schwartz, D. , & Giraud, A. L. (2011). Altered low‐gamma sampling in auditory cortex accounts for the three main facets of dyslexia. Neuron, 72(6), 1080–1090. 10.1016/j.neuron.2011.11.002 22196341

[ejn15894-bib-0045] Leong, V. , & Goswami, U. (2015). Acoustic‐emergent phonology in the amplitude envelope of child‐directed speech. PLoS ONE, 10(12), 1–37. 10.1371/journal.pone.0144411 PMC4671555 26641472

[ejn15894-bib-0046] Leong, V. , Hämäläinen, J. , Soltész, F. , & Goswami, U. (2011). Rise time perception and detection of syllable stress in adults with developmental dyslexia. Journal of Memory and Language, 64(1), 59–73. 10.1016/j.jml.2010.09.003

[ejn15894-bib-0047] Lizarazu, M. , Lallier, M. , Bourguignon, M. , Carreiras, M. , & Molinaro, N. (2021). Impaired neural response to speech edges in dyslexia. Cortex, 135, 207–218. 10.1016/j.cortex.2020.09.033 33387899

[ejn15894-bib-0048] Lizarazu, M. , Lallier, M. , Molinaro, N. , Bourguignon, M. , Paz‐Alonso, P. M. , Lerma‐Usabiaga, G. , & Carreiras, M. (2015). Developmental evaluation of atypical auditory sampling in dyslexia: Functional and structural evidence. Human Brain Mapping, 36(12), 4986–5002. 10.1002/hbm.22986 26356682 PMC6869042

[ejn15894-bib-0049] Lizarazu, M. , Scotto di Covella, L. , van Wassenhove, V. , Rivière, D. , Mizzi, R. , Lehongre, K. , Hertz‐Pannier, L. , & Ramus, F. (2021). Neural entrainment to speech and nonspeech in dyslexia: Conceptual replication and extension of previous investigations. Cortex, 137, 160–178. 10.1016/j.cortex.2020.12.024 33618156

[ejn15894-bib-0050] Lovio, R. , Halttunen, A. , Lyytinen, H. , Näätänen, R. , & Kujala, T. (2012). Reading skill and neural processing accuracy improvement after a 3‐hour intervention in preschoolers with difficulties in reading‐related skills. Brain Research, 1448, 42–55. 10.1016/j.brainres.2012.01.071 22364735

[ejn15894-bib-0051] Mandke, K. , Flanagan, S. , Macfarlane, A. , Gabrielczyk, F. , Wilson, A. , Gross, J. , & Goswami, U. (2022). Neural sampling of the speech signal at different timescales by children with dyslexia. NeuroImage, 253(March), 119077. 10.1016/j.neuroimage.2022.119077 35278708

[ejn15894-bib-0052] Menell, P. , McAnally, K. I. , & Stein, J. F. (1999). Psychophyisical sensitivity and physiological response to amplitude modulation in adult dyslexic listeners. Journal of Speech Language and Hearing Research, 42(4), 797–803. 10.1044/jslhr.4204.797 10450901

[ejn15894-bib-0053] Molinaro, N. , Lizarazu, M. , Lallier, M. , Bourguignon, M. , & Carreiras, M. (2016). Out‐of‐synchrony speech entrainment in developmental dyslexia. Human Brain Mapping, 37, 2767–2783. 10.1002/hbm.23206 27061643 PMC6867425

[ejn15894-bib-0054] National Institute of Child Health and Human Development . (2000). Teaching children to read: An evidence‐based assessment of the scientific research literature on reading and its implications for reading instruction. In *US Government Printing Office*.

[ejn15894-bib-0055] Ozernov‐Palchik, O. , & Gaab, N. (2016). Tackling the “dyslexia paradox”: Reading brain and behavior for early markers of developmental dyslexiax. Wiley Interdisciplinary Reviews: Cognitive Science, 7(2), 156–176. 10.1177/002221949703000208 26836227 PMC4761294

[ejn15894-bib-0056] Peelle, J. E. , & Davis, M. H. (2012). Neural oscillations carry speech rhythm through to comprehension. Frontiers in Psychology, 3(SEP), 1–17. 10.3389/fpsyg.2012.00320 22973251 PMC3434440

[ejn15894-bib-0057] Peterson, R. L. , & Pennington, B. F. (2012). Seminar: Developmental dyslexia. Lancet, 379(9830), 1997–2007. 10.1016/S0140-6736(12)60198-6.Seminar 22513218 PMC3465717

[ejn15894-bib-0058] Peterson, R. L. , & Pennington, B. F. (2015). Developmental dyslexia. Annual Review of Clinical Psychology, 11, 283–307. 10.1146/annurev-clinpsy-032814-112842 25594880

[ejn15894-bib-0059] Picton, T. W. , John, M. S. , Dimitrijevic, A. , & Purcell, D. (2003). Human auditory steady‐state responses. International Journal of Audiology, 42(4), 177–219. 10.3109/14992020309101316 12790346

[ejn15894-bib-0060] Poelmans, H. , Luts, H. , Vandermosten, M. , Boets, B. , Ghesquiere, P. , & Wouters, J. (2011). Reduced sensitivity to slow‐rate dynamic auditory information in children with dyslexia. Research in Developmental Disabilities, 32(6), 2810–2819. 10.1016/j.ridd.2011.05.025 21645986

[ejn15894-bib-0061] Poelmans, H. , Luts, H. , Vandermosten, M. , Boets, B. , Ghesquiére, P. , & Wouters, J. (2012). Auditory steady state cortical responses indicate deviant phonemic‐rate processing in adults with dyslexia. Ear and Hearing, 2002, 134–143. 10.1097/AUD.0b013e31822c26b9 21844810

[ejn15894-bib-0062] Poeppel, D. (2003). The analysis of speech in different temporal integration windows: Cerebral lateralization as “asymmetric sampling in time”. Speech Communication, 41(1), 245–255. 10.1016/S0167-6393(02)00107-3

[ejn15894-bib-0063] Power, A. J. , Colling, L. J. , Mead, N. , Barnes, L. , & Goswami, U. (2016). Neural encoding of the speech envelope by children with developmental dyslexia. Brain and Language, 160, 1–10. 10.1016/j.bandl.2016.06.006 27433986 PMC5108463

[ejn15894-bib-0064] R Core Team . (2018). R: A language and environment for statistical computing. R Foundation for Statistical Computing, Vienna, Austria. https://www.R-project.org/

[ejn15894-bib-0065] Rastle, K. , Lally, C. , Davis, M. H. , & Taylor, J. S. H. (2021). The dramatic impact of explicit instruction on learning to read in a new writing system. Psychological Science, 32(4), 471–484. 10.1177/0956797620968790 33634711 PMC13021060

[ejn15894-bib-0066] Raven, J. C. , Court, J. H. , & Raven, J. (1984). Manual for Raven's progressive matrices and vocabulary scales. Lewis.

[ejn15894-bib-0067] Richardson, U. , & Lyytinen, H. (2014). The GraphoGame method: The theoretical and methodological background of the technology‐enhanced learning environment for learning to read. Human Technology: An Interdisciplinary Journal on Humans in ICT Environments, 10(1), 39–60. 10.17011/ht/urn.201405281859

[ejn15894-bib-0068] Richardson, U. , Thomson, J. M. , Scott, S. K. , & Goswami, U. (2004). Auditory processing skills and phonological representation in dyslexic children. Dyslexia, 10(3), 215–233. 10.1002/dys.276 15341199

[ejn15894-bib-0069] Snowling, M. J. (2000). Dyslexia (2nd ed.). Blackwell.

[ejn15894-bib-0070] Snowling, M. J. , & Hulme, C. (2011). Evidence‐based interventions for reading and language difficulties: Creating a virtuous circle. British Journal of Educational Psychology, 81(1), 1–23. 10.1111/j.2044-8279.2010.02014.x 21391960

[ejn15894-bib-0071] Soltész, F. , Szucs, D. , Leong, V. , White, S. , & Goswami, U. (2013). Differential entrainment of Neuroelectric Delta oscillations in developmental dyslexia. PLoS ONE, 8(10), e76608. 10.1371/journal.pone.0076608 24204644 PMC3799758

[ejn15894-bib-0072] The MathWorks Inc . (2016). MATLAB and Statistics Toolbox Release 2016b. Natick, Massachusetts.

[ejn15894-bib-0073] van den Bos, K. P. , Spelberg, H. C. L. , Scheepstra, A. J. M. , & De Vries, J. R. (1994). De Klepel. Vorm A en B. Een test voor de leesvaardigheid van pseudowoorden. Verantwoording, handleiding, diagnostiek en behandeling.

[ejn15894-bib-0074] Van Herck, S. , Economou, M. , Vanden Bempt, F. , Ghesquière, P. , Vandermosten, M. , & Wouters, J. (2022). Pulsatile modulation greatly enhances neural synchronization at syllable rate in children. Manuscript Submitted for Publication. 10.1016/j.neuroimage.2023.120223 37315772

[ejn15894-bib-0075] Van Herck, S. , Vanden Bempt, F. , Economou, M. , Vanderauwera, J. , Glatz, T. , Dieudonné, B. , Vandermosten, M. , Ghesquière, P. , & Wouters, J. (2022). Ahead of maturation: Enhanced speech envelope training boosts rise time discrimination in pre‐readers at cognitive risk for dyslexia. Developmental Science, 25(3), 1–12. 10.1111/desc.13186 34743382

[ejn15894-bib-0076] Van Hirtum, T. , Ghesquière, P. , & Wouters, J. (2019). Atypical neural processing of rise time by adults with dyslexia. Cortex, 3, 128–140. 10.1016/J.CORTEX.2018.12.006 30640141

[ejn15894-bib-0077] Van Hirtum, T. , Ghesquière, P. , & Wouters, J. (2021). A bridge over troubled listening: Improving speech‐in‐noise perception by children with dyslexia. JARO ‐ Journal of the Association for Research in Otolaryngology, 22, 465–480. 10.1007/s10162-021-00793-4 33861393 PMC8329145

[ejn15894-bib-0078] Van Hirtum, T. , Moncada‐Torres, A. , Ghesquière, P. , & Wouters, J. (2019). Speech envelope enhancement instantaneously effaces atypical speech perception in dyslexia. Ear and Hearing, 1, 1242–1252. 10.1097/aud.0000000000000706 30844835

[ejn15894-bib-0079] Van Phan, T. , Sima, D. , Smeets, D. , Ghesquière, P. , Wouters, J. , & Vandermosten, M. (2021). Structural brain dynamics across reading development: A longitudinal MRI study from kindergarten to grade 5. Human Brain Mapping, 42, 4497–4509. 10.1002/hbm.25560 34197028 PMC8410537

[ejn15894-bib-0093] Van Wieringen, A. , & Wouters, J. (2008). LIST and LINT: Sentences and numbers for quantifying speech understanding in severely impaired listeners for Flanders and the Netherlands. International Journal of Audiology, 47(6), 348e355. 10.1080/14992020801895144 18569107

[ejn15894-bib-0080] Vanden Bempt, F. , Economou, M. , Dehairs, W. , & Vandermosten, M. (2022). Feasibility, enjoyment, and language comprehension impact of a tablet‐ and GameFlow‐based story‐listening game for kindergarteners: Methodological and mixed methods study. JMIR Serious Games, 10(1), 1–26. 10.2196/34698 PMC8987971 35319480

[ejn15894-bib-0081] Vanden Bempt, F. , Economou, M. , Van Herck, S. , Vanderauwera, J. , Glatz, T. , Vandermosten, M. , Wouters, J. , & Ghesquière, P. (2021). Digital game‐based phonics instruction promotes print knowledge in pre‐readers at cognitive risk for dyslexia. Frontiers in Psychology, 12(September), 1–18. 10.3389/fpsyg.2021.720548 PMC8455992 34566803

[ejn15894-bib-0082] Vanden Bempt, F. , Van Herck, S. , Economou, M. , Vanderauwera, J. , Vandermosten, M. , Wouters, J. , & Ghesquière, P. (2022). Speech perception deficits and the effect of envelope‐enhanced story listening combined with phonics intervention in pre‐readers at risk for dyslexia. Frontiers in Psychology, 13, 1021767. 10.3389/fpsyg.2022.1021767 36389538 PMC9650384

[ejn15894-bib-0083] Vanvooren, S. , Poelmans, H. , Hofmann, M. , Ghesquiere, P. , & Wouters, J. (2014). Hemispheric asymmetry in auditory processing of speech envelope modulations in Prereading children. Journal of Neuroscience, 34(4), 1523–1529. 10.1523/JNEUROSCI.3209-13.2014 24453339 PMC6705306

[ejn15894-bib-0084] Vanvooren, S. , Hofmann, M. , Poelmans, H. , Ghesquière, P. , & Wouters, J. (2015). Theta, beta and gamma rate modulations in the developing auditory system. Hearing Research, 327, 153–162. 10.1016/j.heares.2015.06.011 26117409

[ejn15894-bib-0085] Vanvooren, S. , Poelmans, H. , De Vos, A. , Ghesquière, P. , & Wouters, J. (2017). Do prereaders' auditory processing and speech perception predict later literacy? Research in Developmental Disabilities, 70, 138–151. 10.1016/j.ridd.2017.09.005 28938227

[ejn15894-bib-0086] Vellutino, F. R. , Fletcher, J. M. , Snowling, M. J. , & Scanlon, D. M. (2004). Specific reading disability (dyslexia): What have we learned in the past four decades? Journal of Child Psychology and Psychiatry, 45(1), 2–40. 10.1046/j.0021-9630.2003.00305.x 14959801

[ejn15894-bib-0087] Verhoeven, L. (1995). *Drie‐Minuten‐Toets. Handleiding, kaarten en formulieren*. CITO (Centraal Instituut voor Toetsontwikkeling).

[ejn15894-bib-0088] Verwimp, C. , Vanden Bempt, F. , Kellens, S. , Economou, M. , Vandermosten, M. , Wouters, J. , Ghesquière, P. , & Vanderauwera, J. (2020). Pre‐literacy heterogeneity in Dutch‐speaking kindergartners: Latent profile analysis. Annals of Dyslexia, 70, 275–294. 10.1007/s11881-020-00207-9 33074483

[ejn15894-bib-0089] Wanzek, J. , & Vaughn, S. (2007). Research‐based implications from extensive early reading interventions. School Psychology Review, 36(4), 541–561. 10.1080/02796015.2007.12087917

[ejn15894-bib-0090] Zatorre, R. J. , & Belin, P. (2001). Spectral and temporal processing in human auditory cortex. Cerebral Cortex, 11(10), 946–953. 10.1093/cercor/11.10.946 11549617

